# Supplementary material for: Detection of specific uncultured bacteriophages by fluorescence in situ hybridisation in pig microbiome
Source: PLoS One. 2023 Mar 30;18(3):e0283676. doi: 10.1371/journal.pone.0283676 (PMC10062541; doi:10.1371/journal.pone.0283676)
Supplement: S1 File — (PDF) [file pone.0283676.s001.pdf]

FEB 02, 2023

PhageFISH

Line Jensen Ostenfeld[1], Saria Otani[1]

[1]DTU

Saria Otani

ABSTRACT

This is a collection of protocols for phageFISH.

ATTACHMENTS

627-1301.docx

OPEN ACCESS

DOI:
dx.doi.org/10.17504/protocols.io.rm7vzb7z2vx1/v1

Collection Citation: Line Jensen Ostenfeld, Saria Otani 2023. PhageFISH. **protocols.io** https://dx.doi.org/10.17504/protocols.io.rm7vzb7z2vx1/v1

License: This is an open access collection distributed under the terms of the Creative Commons Attribution License, which permits unrestricted use, distribution, and reproduction in any medium, provided the original author and source are credited

Protocol status: Working
We use this collection and it's working

Created: Jan 27, 2023

Last Modified: Feb 02, 2023

COLLECTION integer ID: 75974

Keywords: PhageFISH

ATTACHMENTS

627-1301.docx

FILES

Protocol

NAME

Preparation of Buffers for PhageFISH protocol

VERSION 1

CREATED BY

Saria Otani

OPEN →

Protocol

NAME

PhageFISH for DIG-labelled bacterial probes

VERSION 1

CREATED BY

Saria Otani

OPEN →

Protocol

NAME

PhageFISH detailed protocol

VERSION 1

CREATED BY

Saria Otani

OPEN →

PhageFISH detailed protocol

In 1 collection

Line Jensen Ostenfeld[1], Saria Otani[1]

[1]DTU

Saria Otani

FEB 01, 2023

OPEN ACCESS

DOI:
dx.doi.org/10.17504/protocols.io.4r3l273wqg1y/v1

Protocol Citation: Line Jensen Ostenfeld, Saria Otani 2023. PhageFISH detailed protocol. **protocols.io** https://dx.doi.org/10.17504/protocols.io.4r3l273wqg1y/v1

License: This is an open access protocol distributed under the terms of the Creative Commons Attribution License, which permits unrestricted use, distribution, and reproduction in any medium, provided the original author and source are credited

Protocol status: Working
We use this protocol and it's working

Created: Jan 27, 2023

Last Modified: Feb 01, 2023

PROTOCOL integer ID: 75967

Keywords: Staining and embedding, CARD amplification, Phage probe hybridisation, Antibody binding

ABSTRACT

This protocol details about PhageFISh protocol.

ATTACHMENTS

627-1301.docx

Controls to consider:

- Faecal sample with no target for the phage probe

Timeframe:

**Day 1**		**3h20m**
Prepare samples	**30 minutes***	
Fix samples	**1 hour + 10 min** (1h incubation)	
Prepare permeabilisation buffer and HCl	*10-15 min*	
Prepare ice for permeabilisation	*5 min*	
Wash	**5 min**	❄
Permeabilise cells	**1 hour + 10 min** (1h incubation)	
Wash	**10 min**	
Inactivate peroxidases	**15 min** (10 min incubation)	
Wash	**5 min**	❄

**Day 2**		**6h5m**
Prepare probes	**15 min**	
Hybridisation of cyanine-labelled probes	**3 hours + 10 min** (3h incubation)	
Wash	**20 min** (15 min incubation)	❄
Pre-hybridisation of DIG-labelled probes	**1 hour + 15 min** (1h incubation)	
Prepare probes	*20 min*	
Hybridisation of DIG-labelled probes	**1 hour + overnight** (1h incubation)	☽
**Day 3**		**5h35m**
Wash	**2 hours + 15 min** (30min + 1.5h incubation)	
Prepare antibody washing and CARD buffers	*30 min*	
Antibody binding	**2 hours + 15 min** (30min + 1.5h incubation)	
Wash	**35 min**	
CARD amplification	**1 hour** (45 min incubation)	
Wash	**30 min**	❄
**Day 4**		**1-6h**
Staining and sealing slides	**1 hour***	❄
Microscopy	**1-5 hours***	
**Total:**	**17 hours (not incl. microscopy)**<br>(approx. 12 hours incubation time)<br>**3-5 days**	

***depending on number of samples**

❄ Freezing and stopping possible after step

☽ Overnight incubation after step

MATERIALS

Necessary materials:

- Poly-L-lysine coated glass slides with writing area
- Pencil for writing (DO NOT use sharpie)
- Pipette tip lids for holding glass slides (one will fit four slides, collect one lid for each condition tested)
- Humidity chambers (one for each formamide concentration used simultaneously). Anaerobic growth chambers work well.
- Aluminium foil (to protect samples from light)
- Ice
- Fume hood
- Incubator set to 46 °C
- Incubator (or oven) set to 85 °C
- Water bath set to 48 °C
- Optimised and diluted Cy-labelled probes (see *Optimisation of formamide concentration*)
- Diluted phage probes (see *Buffers and Reagents*)
- All buffers (see *Buffers and Reagents*)
- Faecal samples of interest

Note

- If possible, samples should be submerged in plenty of buffer. Four slides can be submerged in 30-50ml in a pipette tip lid. For washing, very light agitation could be used (e.g. the shaking incubator set to 25rpm).
- For valuable solutions (like probe-solutions), only cover the sample area and handle with care. Use 500µl-1ml to cover sample area.
- All incubations are at room temperature unless specified.
- DO NOT allow samples to dry unless specified.
- When working with paraformaldehyde and formamide always work in the hood.
- After using humidity chambers, allow fumes to evaporate in fume hood overnight.

BEFORE START INSTRUCTIONS

Prepare buffers (see **Preparation of Buffers for PhageFISH protocol**).

Fix faecal samples to glass slides

1 Mix a loopful faecal sample with 10-20 µL PBS (1X) and vortex thoroughly.

2 Allow suspension to settle for 00:05:00 to avoid large debris. 5m

3 Take 10 µL of the supernatant and place on coated glass slide.

4 Smear the droplet thinly over the slide using a cover slip.

Note

Avoid smearing all the way to the edges.

5 Allow the sample to dry – this should not take more than 00:10:00 . 10m

Note

If not dry after 10 minutes, aspirate off excess liquid.

6 Work in fume hood. Overlay the slides with 1% paraformaldehyde (PFA). Ensure the whole sample area is covered (approx. 1 mL).

7 Incubate for 01:00:00 at Room temperature in the fume hood. 1h

Note

This incubation should NOT exceed 01:00:00 !

8 Aspirate off excess PFA.

9 Wash in PBS for 00:01:00 . 1m

Note

If a lot of PFA remains on the sample, rinse twice in PBS.

FREEZING POINT – if necessary, samples can be rinsed in sterile water and 96% ethanol and air dried before freezing in closed box covered with aluminium foil at -20 °C .

Permeabilise cells

10 Add lysozyme to permeabilisation buffer.

11 Overlay samples with permeabilisation buffer.

12 Incubate On ice for 01:00:00 . 1h

13 Discard permeabilisation buffer.

14 Wash samples in PBS for 00:05:00 . 5m

15 Wash samples in sterile water for 00:01:00 . 1m

Inactivate peroxidases

16 Incubate samples in 0.01 Molarity (M) HCl for 00:10:00 . 10m

17 Wash samples in PBS for 00:05:00 . 5m

18 Wash samples in sterile water for 00:01:00 . 1m

19 Wash samples in 96% ethanol for 00:01:00 . 1m

20 Allow slides to dry on blotting paper or filter paper.

Note

FREEZING POINT – if necessary, samples can be frozen after drying. Store in closed container covered with aluminium foil at -20 °C .

Cy-labelled probe hybridisation (16S rRNA probes)

21 Work in fume hood. Place a paper towel in the bottom of the hybridisation chamber and soak in formamide/milliQ solution corresponding to the hybridisation buffer concentration.

22 Overlay samples with hybridisation buffer-probe mix at 0.5 ng/µl of each probe and close humidity chamber.

23 Incubate at 46 °C for 03:00:00 . 3h

24 Prepare the washing buffer – heat to 48 °C .

25 Work in fume hood. Overlay the samples with washing buffer and incubate for 00:15:00 at 48 °C (in humidity chamber to avoid formamide fumes). 15m

26 Wash samples in sterile water.

27 Allow samples to dry.

Note

FREEZING POINT – if necessary, samples can be frozen after drying. Store in closed container covered with aluminium foil at -20 °C .

Phage probe hybridisation

28 Work in fume hood. Place a paper towel in the bottom of the hybridisation chamber and soak in formamide/milliQ solution corresponding to the hybridisation buffer concentration.

29 Overlay samples with hybridisation buffer (no probes!) and close humidity chamber (500 µL per slide).

30 Incubate for 01:00:00 at 46 °C . 1h

31 Cover the samples with hybridisation buffer-probe mix at 10 pg/µl of each probe (500µl per slide).

32 Place the dish back in the humidity chamber and incubate for 01:00:00 at 85 °C . 1h

33 Immediately place the humidity chamber at hybridisation temperature Overnight . 1h

34 Wash slides.

34.1 Wash slides in gene washing buffer I for 00:01:00 . (1/3) 1m

34.2 Wash slides in gene washing buffer I for 00:01:00 . (2/3) 1m

34.3 Wash slides in gene washing buffer I for 00:01:00 . (3/3) 1m

34.4 Wash slides in gene washing buffer I for 00:30:00 at 42 °C . 30m

35 Wash slides.

35.1 Wash slides in gene washing buffer II for 00:01:00 . (1/3) 1m

35.2 Wash slides in gene washing buffer II for 00:01:00 . (2/3) 1m

35.3 Wash slides in gene washing buffer II for 00:01:00 . (3/3) 1m

35.4 Wash slides in gene washing buffer II for 01:30:00 at 42 °C . 1h 30m

36 Wash slides in PBS for 00:01:00 . 1m

Antibody binding

37 Cover slides with antibody-blocking solution. Incubate for 00:30:00 . 30m

38 Discard antibody-blocking solution and cover with antibody binding solution. Incubate for 01:30:00 . 1h 30m

39 Wash slides.

39.1 Wash slides in antibody washing solution for 00:01:00 . 1m

39.2 Wash slides in antibody washing solution for 00:10:00 . (1/3) 10m

39.3 Wash slides in antibody washing solution for 00:10:00 . (2/3) 10m

39.4 Wash slides in antibody washing solution for 00:10:00 . (3/3) 10m

CARD amplification

40 Mix 1 mL amplification buffer with 10 µL H_2O_2 and 2 µL Alexa tyramides (488). Vortex to mix.

41 Cover slides with CARD buffer-tyramide mix (approx. 500 µL per slide). Incubate at 37 °C for 00:45:00 . 45m

42 Wash slides.

42.1 Wash slides in PBS for 00:01:00 . 1m

42.2 Wash slides in PBS for 00:05:00 . 5m

42.3 Wash slides in PBS for 00:10:00 at 46 °C . 10m

42.4 Wash slides in PBS for 00:10:00 at 46 °C . 10m

43 Wash slides in sterile water for 00:01:00 . 1m

44 Wash slides in 96% ethanol for 00:01:00 . 1m

Note

FREEZING POINT

Staining and embedding

45 Mix 1 mL SlowFade Gold antifade reagent with 1 5m/ml DAPI (final concentration 5 µg/mL , can be stored at Room temperature).

46 Place 10 µL solution in small droplets on the slides.

47 Place coverslip and press down gently to remove air pockets without disturbing the sample area.

48 Seal edges with clear nail polish.

49 Samples can now be stored at -20 °C in covered container indefinitely.

FEB 01, 2023

OPEN ACCESS

DOI:
dx.doi.org/10.17504/protocols.io.kqdg3931pg25/v1

Protocol Citation: Line Jensen Ostenfeld, Saria Otani 2023. PhageFISH for DIG-labelled bacterial probes. **protocols.io** https://dx.doi.org/10.17504/protocols.io.kqdg3931pg25/v1

License: This is an open access protocol distributed under the terms of the Creative Commons Attribution License, which permits unrestricted use, distribution, and reproduction in any medium, provided the original author and source are credited

Protocol status: Working
We use this protocol and it's working

Created: Jan 25, 2023

Last Modified: Feb 01, 2023

PROTOCOL integer ID: 75844

Keywords: PhageFISH, DIG-labelled bacterial probes

PhageFISH for DIG-labelled bacterial probes

In 1 collection

Line Jensen Ostenfeld[1], Saria Otani[1]

[1]DTU

Saria Otani

ABSTRACT

This protocol details about PhageFISH for DIG-labelled bacterial probes.

ATTACHMENTS

627-1301.docx

MATERIALS

Reagents

- 1% paraformaldehyde
- PBS
- 0.01M HCl
- sterile water
- 96% ethanol
- permeabilisation buffer
- hybridisation buffer
- gene washing buffer I
- gene washing buffer II
- amplification buffer
- Alexa tyramides (488)
- Tris-HCl
- RNase I
- RNase A
- antibody-blocking solution
- antibody binding solution
- antibody washing solution
- Alexa tyramides (594)
- SlowFade Gold
- DAPI dye

Fix liquid samples to glass slides

1 Place liquid sample in a 30-50 µL droplet on poly-L-lysine coated slide.

2 Dry in warm incubator for approx. 00:30:00 or until the droplet has dried out. 30m

3 OPTIONAL: if sample is very dilute add several droplets and repeat drying procedure.

4 Add 1% paraformaldehyde to cover the sample area.

5 Incubate at Room temperature for 01:00:00 . 1h

6 Aspirate the paraformaldehyde off.

7 Rinse samples in PBS for 00:01:00 . 1m

Fix faecal samples to glass slides

8 Mix a small faecal sample with 10-20 µL PBS (1X) and vortex thoroughly.

9 Allow suspension to settle for 00:05:00 . 5m

10 Take 10 µL of the supernatant and place on coated glass slide.

11 Smear the droplet over the slide using a cover slip.

12 Allow the sample to dry – this should not take more than 00:10:00 . 10m

13 Overlay the slides with 1% paraformaldehyde. Ensure the whole sample area is covered (approx. 1 mL).

14 Incubate for 01:00:00 at Room temperature or Overnight at 4 °C . 1h

15 Aspirate off excess paraformaldehyde.

16 Wash in PBS for 00:01:00 . 1m

Note

FREEZING POINT

Permeabilise cells

17 Add lysozyme to permeabilisation buffer.

18 Overlay samples with permeabilisation buffer.

19 Incubate On ice for 01:00:00 . 1h

20 Wash samples in PBS for 00:05:00 . 5m

21 Wash samples in sterile water for 00:01:00 . 1m

Inactivate peroxidases

22 Incubate samples in 0.01 Molarity (M) HCl for 00:10:00 . 10m

23 Wash samples in PBS for 00:05:00 . 5m

24 Wash samples in sterile water for 00:01:00 . 1m

25 Wash samples in 96% ethanol for 00:01:00 . 1m

26 Allow slides to dry on blotting paper or filter paper.

27 Place filters in a petri dish and spot up to 100 µL hybridisation buffer to cover the filters.

28 Transfer to a humidity chamber with hybridisation buffer soaked paper towels.

29 Incubate for 01:00:00 at hybridisation temperature______. 1h

30 Mix 1 mL gene hybridisation buffer with 1 µL of each probe. Vortex to mix.

31 Place one droplet of 30-100 µL probe mix on a petri dish for each filter.

32 Place the filters face down in the probe mix droplets.

33 Place the dish back in the humidity chamber and incubate for 01:00:00 at 85 °C . 1h

34 Immediately place the humidity chamber at hybridisation temperature Overnight . 1h

35 Wash filters.

35.1 Wash filters in gene washing buffer I 00:01:00 . (1/3) 1m

35.2 Wash filters in gene washing buffer I 00:01:00 . (2/3) 1m

35.3 Wash filters in gene washing buffer I 00:01:00 . (3/3) 1m

35.4 Wash filters in gene washing buffer I 00:30:00 at 42 °C . 30m

36 Wash filters.

36.1 Wash filters in gene washing buffer II for 00:01:00 . (1/3) 1m

36.2 Wash filters in gene washing buffer II for 00:01:00 . (2/3) 1m

36.3 Wash filters in gene washing buffer II for 00:01:00 . (3/3) 1m

36.4 Wash filters in gene washing buffer II for 01:30:00 at 42 °C . 1h 30m

37 Wash filters in PBS for 00:01:00 . 1m

Antibody binding

38 Place filters in a petri dish and add antibody blocking solution to cover the filters. Incubate for 00:30:00 . 30m

39 Move filters to antibody binding solution and incubate for 01:30:00 . 1h 30m

40 Wash filters.

40.1 Wash filters in antibody washing solution for 00:01:00 . 1m

40.2 Wash filters in antibody washing solution for 00:10:00 . (1/3) 10m

40.3 Wash filters in antibody washing solution for 00:10:00 . (2/3) 10m

40.4 Wash filters in antibody washing solution for 00:10:00 . (3/3) 10m

CARD amplification

41 Mix 1 mL amplification buffer with 10 µL H_2O_2 and 2 µL Alexa tyramides (488). Vortex to mix.

42 Place filters in a petri dish and cover with probe mix by spotting droplets of 30-100 µL .

43 Wash filters.

43.1 Wash filters in PBS for 00:01:00 1m

43.2 Wash filters in PBS for 00:05:00 . 5m

43.3 Wash filters in PBS for 00:10:00 at 46 °C . (1/2) 10m

43.4 Wash filters in PBS for 00:10:00 at 46 °C . (2/2) 10m

44 Wash filters in sterile water for 00:01:00 . 1m

45 Wash filters in 96% ethanol for 00:01:00 . 1m

Remove RNases

46 Add 10.8 mL sterile water, 1.2 mL Tris-HCl (1M, pH 8), 15 µL RNase I, and 30 µL RNase A to a 15ml falcon tube.

47 Place filters in the RNase solution and incubate for 01:00:00 at 37 °C . 1h

48 Wash filters in PBS for 00:05:00 . 5m

49 Repeat wash.

50 Wash filters in sterile water for 00:01:00 . 1m

Gene hybridisation

51 Cover samples with hybridisation buffer.

52 Transfer to a humidity chamber with formamide soaked paper towels at the corresponding concentration.

53 Incubate for 01:00:00 at hybridisation temperature (approx. 46 °C). 1h

54 Mix 1 mL gene hybridisation buffer with 1 µL of each probe. Vortex to mix.

55 Cover the samples with the hybridisation buffer-probe mix.

56 Place the dish back in the humidity chamber and incubate for 01:00:00 at 85 °C . 1h

57 Immediately place the humidity chamber at hybridisation temperature Overnight . 1h

Note

OVERNIGHT

58 Wash filters.

58.1 Wash filters in gene washing buffer I for 00:01:00 . (1/3) 1m

58.2 Wash filters in gene washing buffer I for 00:01:00 . (2/3) 1m

58.3 Wash filters in gene washing buffer I for 00:01:00 . (3/3) 1m

58.4 Wash filters in gene washing buffer I for 00:30:00 at 42 °C . 30m

59 Wash filters.

59.1 Wash filters in gene washing buffer II for 00:01:00 . (1/3) 1m

59.2 Wash filters in gene washing buffer II for 00:01:00 . (2/3) 1m

59.3 Wash filters in gene washing buffer II for 00:01:00 .(3/3) 1m

59.4 Wash filters in gene washing buffer II for 01:30:00 at 42 °C . 1h 30m

60 Wash filters in PBS for 00:01:00 . 1m

Antibody binding

61 Place filters in a petri dish and add antibody-blocking solution to cover the filters. Incubate for 00:30:00 . 30m

62 Move filters to antibody binding solution and incubate for 01:30:00 . 1h 30m

63 Wash filters.

63.1 Wash filters in antibody washing solution for 00:01:00 . 1m

63.2 Wash filters in antibody washing solution for 00:10:00 . (1/3) 10m

63.3 Wash filters in antibody washing solution for 00:10:00 . (2/3) 10m

63.4 Wash filters in antibody washing solution for 00:10:00 . (3/3) 10m

CARD amplification

64 Mix 1 mL amplification buffer with 10 µL H_2O_2 and 2 µL Alexa tyramides (594). Vortex to mix.

65 Place filters in a petri dish and cover with probe mix by spotting droplets of 30-100 µL . Incubate at 37 °C for 00:45:00 . 45m

66 Wash filters.

66.1 Wash filters in PBS for 00:01:00 . 1m

66.2 Wash filters in PBS for 00:05:00 . 5m

66.3 Wash filters in PBS for 00:10:00 at 46 °C . 10m

66.4 Wash filters in PBS for 00:10:00 at 46 °C . 10m

67 Wash filters in sterile water for 00:01:00 . 1m

68 Wash filters in 96% ethanol for 00:01:00 . 1m

Note

OPTIONAL FREEZING POINT

Staining

69 Mix 1 mL SlowFade Gold with 1 µL 5 mg/mL DAPI dye.

70 Apply 5-10 µL mix in droplets to each slide.

71 Apply coverglass and carefully press down to seal sample with minimal air bubbles.

72 Seal with clear nail polish on all edges of the sample.

73 Allow to cure completely.

74 Store at -20 °C .

Preparation of Buffers for PhageFISH protocol

In 1 collection

Line Jensen Ostenfeld[1], Saria Otani[1]

[1]DTU

Saria Otani

FEB 01, 2023

OPEN ACCESS

DOI:
dx.doi.org/10.17504/protocols.io.dm6gpjop8gzp/v1

Protocol Citation: Line Jensen Ostenfeld, Saria Otani 2023. Preparation of Buffers for PhageFISH protocol. **protocols.io** https://dx.doi.org/10.17504/protocols.io.dm6gpjop8gzp/v1

License: This is an open access protocol distributed under the terms of the Creative Commons Attribution License, which permits unrestricted use, distribution, and reproduction in any medium, provided the original author and source are credited

Protocol status: Working
We use this protocol and it's working

Created: Jan 27, 2023

Last Modified: Feb 01, 2023

PROTOCOL integer ID: 75961

Keywords: Permeabilisation buffer, rRNA hybridisation buffer, rRNA hybridisation wash buffer, rRNA CARD buffer, Gene hybridisation buffer, Gene hybridisation wash buffer

ABSTRACT

This protocol details about preparation of various buffers for PhageFISH protocol.

ATTACHMENTS

627-1301.docx

MATERIALS

Materials and Reagents

- PBS [pH 7.5] (10 x)
- Tris-HCl [pH 8.0](1 M)
- EDTA (0.5)
- Water
- Lysozyme
- Dextran sulphate
- NaCl (5 M)
- Tris-HCl [pH 8.0] (1 M)
- Nucleic acid blocking reagent (10%)
- Sheared salmon sperm (10 mg/ml)
- Yeast RNA (10 mg/ml)
- Formamide (100%)
- SDS (20%)
- SSC (20 x)
- 0.22µm syringe filter
- 50 ml falcon tube

Permeabilisation buffer

1 50 ml:

A	B
PBS [pH 7.5] (10 x)	5 ml
Tris-HCl [pH 8.0] (1 M)	5 ml
EDTA (0.5)	5 ml
Water	35 ml
Lysozyme	

Permeabilisation mix: Mix PBS, Tris-HCl, EDTA, and water.

2 High conc. lysozyme buffer: Dissolve lysozyme in appropriate buffer volume (50 mg lysozyme to 10 mL buffer). It may be necessary to heat the solution to 37 °C .

3 Dilute lysozyme buffer into large buffer volume by adding 1 part lysozyme buffer to 9 parts permeabilisation mix.

4 Final concentration:

A	B
PBS	1 x
Tris-HCl	0.1 M
EDTA	0.05 M
Lysozyme	0.5 mg/ml

Note

- Permeabilisation buffer should not be stored.
- Prepare in aliquots of 1 mL .

rRNA hybridisation buffer

5 40 ml:

A	B
Dextran sulphate	4 g
NaCl (5 M)	7.2 ml
Tris-HCl [pH 8.0] (1 M)	0.8 ml
Water	4 ml
Nucleic acid blocking reagent (10%)	4 ml

A	B
Sheared salmon sperm (10 mg/ml)	1 ml
Yeast RNA (10 mg/ml)	1 ml
Formamide (100%)	17.5 ml
SDS (20%)	40 µl

Mix dextran sulphate, NaCl, Tris-HCl, and water in a falcon tube and vortex or shake thoroughly to disperse dextran sulphate. Heat solution in waterbath at 37-48 °C and vortex until dextran sulphate is completely dissolved.

6 Cool the solution down to Room temperature .

7 Add nucleic acid blocking agent, sheared salmon sperm, yeast RNA, formamide, and SDS. Adjust volume with water to reach 40 mL if necessary.

8 Vortex to mix.

9 Spin down solution briefly and filter through 0.22µm syringe filter.

10 Final concentration:

A	B
Dextran sulphate	10%
NaCl	0.9 M
Tris-HCl	20 mM
Nucleic acid blocking reagent	1%
Sheared salmon sperm	0.25 mg/ml
Yeast RNA	0.25 mg/ml
Formamide	35%
SDS	0.02%

11 Store in aliquots at -20 °C . Reheat to 37 °C before use to redissolve precipitate.

12 Prepare several in aliquots of 900 µL .

rRNA hybridisation wash buffer

13 50 ml:

	A	B
	*NaCl (5 M)	700 µl
	*EDTA [pH 8.0] (0.5 M)	500 µl
	Tris-HCl (1 M)	1 ml
	Water	up to 50 ml
	SDS (20%)	25 µl

Mix *NaCl, *EDTA, and Tris-HCl in 50 ml falcon tube.

Note

* **NOTE**: Na^+ concentrations depend on the amount of formamide used in the hybridisation buffer. The formamide concentration is calculated based on probe properties to achieve a hybridisation temperature of 42-50 °C .

14 Add water up the 50 ml mark.

15 Add SDS.

16 Final concentrations:

	A	B
	NaCl	70 mM
	EDTA	5 mM
	Tris-HCl	20 mM
	SDS	0.01%

17 The formamide (FA) concentrations and the corresponding Na^+ ions concentrations when washing at 48 °C are as follows:

	A	B
	0% FA	900 mM Na+
	5%FA	636 mM Na+
	10% FA	450 mM Na+
	15% FA	318 mM Na+
	20% FA	225 mM Na+
	25% FA	159 mM Na+
	30% FA	112 mM Na+
	35% FA	80 mM Na+
	40% FA	56 mM Na+
	45% FA	40 mM Na+
	50% FA	28 mM Na+
	55% FA	20 mM Na+
	60% FA	14 mM Na+

18 Prepare in aliquots of 50.

Note

Prepare at least two aliquots per cycle.

rRNA CARD buffer

19 40 ml:

	A	B
	Dextran sulphate	4 g
	PBS [pH 7.4] (10 x)	4 ml
	NaCl (5 M)	16 ml
	Water	up to 40 ml
	Nucleic acid blocking reagent (10%)	400 µl

Mix dextran sulphate, PBS, and NaCl. Add water up to 40 ml. vortex thoroughly to disperse dextran sulphate. Heat solution in waterbath at 37-48 °C and vortex until dextran sulphate is completely dissolved.

20 Allow solution to cool down to room temperature and add nucleic acid blocking reagent.

21 Vortex to mix.

22 Spin down briefly.

23 Filter through 0.22 µm syringe filter.

24 Final concentration:

A	B
PBS	1x
Dextran sulphate	10%
Nucleic acid blocking reagent	0.10%
NaCl	2 M

25 Store in aliquots at 4 °C . Reheat to 37 °C before use to redissolve precipitate.

26 Prepare in aliquot of 3 mL .

Gene hybridisation buffer

27 40 ml:

A	B
Dextran sulphate	4 g
SSC (20 x)	10 ml
EDTA [pH 8.0] (0.5 M)	1.6 ml
Water	4.4 ml
Nucleic acid blocking reagent (10%)	4 ml
Sheared salmon sperm (10 mg/ml)	1 ml
Yeast RNA (10 mg/ml)	1 ml
Formamide (100%)	14 ml
SDS (20%)	200 µl

Mix dextran sulphate, SSC, EDTA, and water in a falcon tube and vortex or shake thoroughly to disperse dextran sulphate. Heat solution in waterbath at 37-48 °C

and vortex until dextran sulphate is completely dissolved.

28 Cool the solution down to Room temperature .

29 Add nucleic acid blocking agent, sheared salmon sperm, yeast RNA, formamide, and SDS.

30 Vortex to mix.

31 Spin down solution briefly and filter through 0.22 µm syringe filter.

32 Final concentration:

A	B
Formamide	35%
SSC	5x
Dextran sulphate	10%
SDS	0.10%
EDTA	20 mM
Nucleic acid blocking reagent	1%
Sheared salmon sperm	0.25 mg/ml
Yeast RNA	0.25 mg/ml

33 Store in aliquots at -20 °C . Reheat to 42 °C before use to redissolve precipitate.

Gene hybridisation wash buffer I

34 50 ml:

A	B
SSC (20 x)	5 ml
SDS	250 µl

A	B
Water	up to 50 ml

Mix SSC and water in a 50 ml falcon tube.

35 Add SDS.

36 Vortex to mix.

37 Final concentration:

A	B
SSC	2 x
SDS	0.1%

38 Store for 1-2 days at 42 °C .

Gene hybridisation wash buffer II

39 50 ml:

A	B
SSC (20 x)	250 µl
SDS	250 µl
Water	up to 50 ml

Mix SSC and water in a 50 ml falcon tube.

40 Add SDS.

41 Vortex to mix.

42 Final concentration:

SSC	0.1 x
SDS	0.10%

43 Store for 1-2 days at 42 °C .

Gene CARD amplification buffer

44 40 ml:

A	B
Dextran sulphate	8 g
PBS [pH 7.4] (10 x)	4 ml
NaCl (5 M)	16 ml
Water	15.6 ml
Nucleic acid blocking reagent (10%)	400 µl

Mix dextran sulphate, PBS, NaCl, and water.

45 Vortex or shake thoroughly to disperse dextran sulphate. Heat solution in waterbath at 37-48 °C and vortex until dextran sulphate is completely dissolved.

46 Allow solution to cool down to room temperature and add nucleic acid blocking reagent.

47 Vortex to mix.

48 Spin down briefly.

49 Filter through 0.22 µm syringe filter.

50 Final concentrations:

	A	B
	PBS	1x
	Dextran sulphate	20%
	Blocking reagent	0.10%
	NaCl	2 M

51 Store in aliquots at 4 °C . Reheat to 37 °C before use to redissolve precipitate.

protocols.io
