# Supplementary material for: Detection of specific uncultured bacteriophages by fluorescence in situ hybridisation in pig microbiome
Source: PLoS One. 2023 Mar 30;18(3):e0283676. doi: 10.1371/journal.pone.0283676 (PMC10062541; doi:10.1371/journal.pone.0283676)
Supplement: S2 File — (DOCX) [file pone.0283676.s002.docx]

Supplementary Information for

Detection of specific uncultured bacteriophages by fluorescence *in situ* hybridisation in pig microbiome

Line Jensen Ostenfeld^a^, Patrick Munk^a^, Frank M. Aarestrup^a^ and Saria Otani^a^

^a^ Research group for Genomic Epidemiology, National Food Institute

# **Material and methods**

## Phage probe hybridisation

For phage gene probe hybridisation, slides were pre-hybridised in 500µl hybridisation buffer II (35% formamide (v/v), 10% (w/v) dextran sulphate, 5x SSC, 20mM EDTA, 1% (w/v) nucleic acid blocking solution, 0.35mg/ml DNA, 0.25mg/ml RNA, and 0.1% (w/v) SDS) at 46°C for 1 hour. 10pg/µl of each phage probe was then added to hybridisation buffer II, and 500µl were added to the slides for a final probe concentration of 5pg/µl in hybridisation buffer II. Probes were denatured at 85°C for 1 hour and hybridised at 46°C overnight. Slides were rinsed and incubated in wash buffer II (2xSSC, 0.1% (w/v) SDS) and wash buffer III (0.1xSSC, 0.1% (w/v) SDS). HRP-linked antibodies were added as a link between DIG and Alexa tyramides by submerging slides in antibody blocking solution (1xPBS, 1x Western Blocking Reagent) for 30 minutes at room temperature, followed by incubation for 1.5 hours in antibody binding solution (0.3U/ml anti-DIG HRP-conjugated antibody, 1xPBS, 1x Western Blocking Reagent). Unbound antibodies were rinsed off in antibody blocking solution. Alexa Fluor tyramides were added for signal amplification in a CARD-step by 45-minute incubation in 500µl CARD buffer (20% (w/v) dextran sulphate, 0.1% (v/v) nucleic acid blocking reagent, 2M NaCl, 0.0015% H_2_O_2_, Alexa Fluor tyramides at concentration recommended by supplier) at 37°C. Sample slides were then rinsed and washed in warm PBS, sterile water, and dried in 96% ethanol before storage at -20°C.

## CARD amplification of 16S bacterial probes

DIG-labelled 16S rRNA probe, Ebac1790 (Supplementary Table S2), was hybridised to a pure culture of either *Escherichia coli 11303* (target) or *Enterococcus faecalis* (clinical isolate, non-target) on glass slides. For pure cultures, bacteria were grown overnight in Lysogeny Broth (*E. coli*) or Brain Heart Infusion broth (*E. faecalis*) at 37°C. 10µl droplets of liquid culture were placed on a glass slide and allowed to dry for 30min at 37°C until the liquid had evaporated. Cells were fixed by covering sample area with 1% (v/v) paraformaldehyde for 1 hour at room temperature and rinsed in PBS. Cells were permeabilised in permeabilisation buffer on ice for 1h, rinsed in PBS for 5min and sterile water for 1min. This was followed by inactivation of peroxidases in 0.01M HCl for 10min and again rinsed in PBS for 5min and sterile water for 1min. Slides were prepared for hybridisation in 500µl hybridisation buffer II in humidity chamber at 46°C for 1h. DIG-labelled 16S rRNA probes were then hybridised to the slides by adding 500µl hybridisation buffer II with 6ng/µl DIG-labelled probes (final concentration 3ng/µl) and incubating at 85°C for 1h followed by incubation at 46°C overnight. Slides were then rinsed in wash buffer II for 3x1min and 30min at 42°C and wash buffer III for 3x1min and 1.5h at 42°C. Anti-DIG antibodies were bound to the slides by immersing in antibody blocking solution (1xPBS, 1% Western Blocking Solution (Roche)) for 30min followed by antibody binding solution (antibody blocking solution plus 0.3U/ml Anti-DIG HRP-conjugated antibody (Roche)) for 1.5h at room temperature. Slides were washed in antibody blocking solution for 3x10min. Signals were amplified by covering the slides in CARD buffer with Alexa Fluor tyramides 488 at 37°C for 45min. Remaining unbound RNA was removed by incubation in RNase solution (0.1U RNase I (Sigma), 75µg/ml RNase A (Invitrogen), 0.1M Tris-HCl pH 8.0 (Invitrogen)) at 37°C for 1h and rinsed in PBS for 5min and sterile water for 1min. Slides were immersed in 0.2M HCl for 10min and rinsed in PBS for 1min and 5min and in sterile water for 1min. For signal detection, slides were embedded in SlowFade Gold reagent (Invitrogen) and DAPI (Roche) and cover slip.

For phage probe signal evaluation, phage probes were hybridised as described above except CARD amplification was carried out using Alexa Fluor 594 tyramides instead of Alexa Fluor 488 tyramides.

DIG-labelled 16S rRNA probe hybridisation of Ebac1790 showed optimal signal intensity at 45% formamide in the hybridisation buffer and resulted in high-resolution images with very clear signals. Subsequent hybridisation of DIG-labelled phage probes, however, quenched the signal. Phage probes had no target in the pure cultures and were not expected to attach to anything. No signals were observed in the visible spectrum of Alexa Fluor 594 (phage).

## Image acquisition and enhancements

As different samples have various background signals, a number of samples have more background fluorescence and a number of dyes appear more intense (such as DAPI), the same exposure time is not applicable to all samples. Increasing exposure time may obscure signal intensity for between-sample comparisons, but will not obscure the level of non-specific binding and background fluorescence compared to the probe signals. Images of signals other than DAPI were captured at the same exposure time for the same sample as far as were possible. When weak or no negative control signal (Cy5) was observed, exposure was increased for negative control only, to ensure all off-target binding and background fluorescence could be assessed.

## Signal strength may depend on target accumulation

Signal strength may increase when several targets are in close vicinity of each other. Organisms encoding multiple copies of the targeted gene could produce a stronger accumulated signal. Phages rarely encode several copies of the same gene due to the spatial constraints of small capsid size. DNA is packaged densely and only minimal redundancy is present. Phages with larger genomes tend to have a larger capsid size, which allows more non-essential genes and sometimes more terminal redundancy ^2–4^. Internalised phages and clustered phages (*i.e.* immediately post burst or aggregating phages) are also expected to produce strong signals as signals from several phages accumulate.

## References

1. Amann RI, Binder BJ, Olson RJ, Chisholm SW, Devereux R, Stahl D. Combination of 16S rRNA-targeted oligonucleotide probes with flow cytometry for analyzing mixed microbial populations. *Appl Environ Microbiol*. 1990;56(6):1919-1925.

2. Yuan Y, Gao M. Jumbo Bacteriophages: An Overview. *Front Microbiol*. 2017;8(403). doi:10.3389/fmicb.2017.00403

3. Hendrix RW. Jumbo Bacteriophages. In: Van Etten JL, ed. *Lesser Known Large DsNDA Viruses. Current Topics in Microbiology and Immunology*. Vol 328. Berlin, Heidelberg: Springer; 2009:229-240.

4. Hua J. Capsid Structure and DNA Packing in Jumbo Bacteriophages. 2016. http://d-scholarship.pitt.edu/27666/4/ETD_JianfeiHua.pdf. Accessed April 9, 2019.

5. Saad AM, Soliman AM, Kawasaki T, et al. Systemic method to isolate large bacteriophages for use in biocontrol of a wide-range of pathogenic bacteria. *J Biosci Bioeng*. 2018;127(1):73-78. doi:10.1016/j.jbiosc.2018.07.001

# **SUPPLEMENTARY TABLES**

## S1 Table: Assembled phage contig size^a^

| Assembled phage | Faecal sample | Genome / Contig size (bp) |
| --- | --- | --- |
| Phage A | F12 | 286,136 |
|  | F33 | 282,976 |
| Phage B | F42 | 250,325 |
|  | F71 | 247,510 |
| Phage C | F67 | 227,104 |
|  | F78 | 207,487 |
| Phage D | F95 | 111,278 |
|  | F101 | 237,543 |
| Phage E | F49 | 239,299 |
|  | F59 | 336,322 |
| *^a^Phage contigs assembled from faecal metagenomes of two individual faecal samples.* | | |

## S2 Table: Probe sequences^a^

| Phage probes | Probe length | Target | Reference | Fluorophore | Visible spectrum | Microscope filter |
| --- | --- | --- | --- | --- | --- | --- |
| DenA1 | 300bp | Phage A | This study | Alexa Fluor 488 | 490/525 | FITC |
| ATTGCAACAAATTGACTTACCGCCGAACATTGAAAATATCCCACCTAGATGTTTTCAAGCTTGTCTTAAGCTGAGAAATCTTAATCTTAATAAAGTTGAAACAATCGAAGAAGAAGCTTTTCAATCATCTGGGATTAGAGTACTAGATATTCCTGTGTCTTTARCAACAGTCGGAAAGAAAGCTTTTGAAGGTTGCATTTATCTTAGCGACATTGTTTGTGAAAGATTTTTGCCTCCTAAACTTTCTGCATCATCTTTTTATGGTTGTTCAATCAAGAATGTTTGGGTTTTCTCAGAAGC | | | | | | |
| DenA2 | 300bp | Phage A | This study | Alexa Fluor 488 | 490/525 | FITC |
| AAGGTGTTGGACTTGTAACTGAGATGTTCAACCCAGGGTGTGATACTGAAGATGAAACTGCTGAAGAGTTTGAATCATCTAATCCAGAGTTTAATAAGCTTAAAAAAGCATTGCTTGAAAGCGACGGTGATAGTGATGATGACAAAGACGATAGTGAAGATGAAGCAGGAGTAGGCGGCGGTTCAGGCGCAGCTGCTGACCAAGCAGATAAAGATGCTGAAGAYCAGATGACTGCAGCAATTAAAGCAGCAAATGCCCAAGAAGCTAAAGCAAAAGAATGTATCGCAAAAGAACTTGGCA | | | | | | |
| DenA3 | 300bp | Phage A | This study | Alexa Fluor 488 | 490/525 | FITC |
| GGGAGATTACCTTTCACAAGCAAAAGCAAAGCGCCAAAAAGAGCTTGATGAACTTAATGCAAAGTTGATTGAAGCCGGTGAAGAAGTAACTCAAACAGAAGCTGATGATACGCGTCTTGTTAAAAATGCATCAGCTACAAATGATAGCGATARGTACTATGATACTTTTGACTTGAATACTTTTAAGGCRGCTCGTGCTTCATTTACATATCGTGCACCTGGCTTCAAAGCAATCAATGATAACATCAATATGATTCAGTGGCTTCTTGAAGGCTGTTCGGGAATCGTTTCAACATATCT | | | | | | |
| DenA4 | 300bp | Phage A | This study | Alexa Fluor 488 | 490/525 | FITC |
| TGCTTGGGTTGGAGCAAAATATTATTTCACATCATATGATATTGCTCCAAGATTTATGTACAATCTTGTTTCAACTCAAAATGGATTCTTAGCAGGCGCAAAAGACTTGCTCTGTGGACATATAAAAGACTCAAAAGGTCGTGATACAGGTAAGTACATAATGTCGTTTGACTATGTTCGTGACATTATGGTAGGTACTAATCGTTCRGCWGCTCTTGTTTCACCTCGTTCATACTTCCTTGCAAATCCTGAAGGAAAGTTTAAGGATAAAGACTCAGGAACAATTTACAACTGCGAAGA | | | | | | |
| DenA5 | 300bp | Phage A | This study | Alexa Fluor 488 | 490/525 | FITC |
| ATTGCAACAAATTGACTTACCGCCGAACATTGAAAATATCCCACCTAGATGTTTTCAAGCTTGTCTTAAGCTGAGAAATCTTAATCTTAATAAAGTTGAAACAATCGAAGAAGAAGCTTTTCAATCATCTGGGATTAGAGTACTAGATATTCCTGTGTCTTTARCAACAGTCGGAAAGAAAGCTTTTGAAGGTTGCATTTATCTTAGCGACATTGTTTGTGAAAGATTTTTGCCTCCTAAACTTTCTGCATCATCTTTTTATGGTTGTTCAATCAAGAATGTTTGGGTTTTCTCAGAAGC | | | | | | |
| DenA6 | 300bp | Phage A | This study | Alexa Fluor 488 | 490/525 | FITC |
| TCACTTCCACCATTTGACATCAACTGGGACAATCTTGCTGCAAGCGAGTCAGGAATCATATTCAACTTGTTTGGCGGTACTGCAGCAGATAAGATTAGAGACCTTACAAAAGCTGGTGTTACAGATCCTCAGGCATTACTTTGTGGAGCTAAACAAAGTACAGACTATTGGAGAATTCCTGTAAGTGGAAAGCTTCCTAAGGCTGAAGGATATAAATCAGATATAACACTTGAGTTCTGCAGTCAAACTGAAAGTGGACTYGTAATGACAATACCTGCTAAAAATCCTGCTGCTTTGTCA | | | | | | |
| DenB1 | 300bp | Phage B | This study | Alexa Fluor 488 | 490/525 | FITC |
| TTATTACGGTCATTTTCTCATCTGCCTGTGATRYCATTTATACTCTCCTCTCTAAATAATTTACTGCTAAGTTTCGACTAAAGTG?AAAAAAAATAAAGCGATGCGAGGAGCCTGAATCCTCGCATCTATATTCWAATTGAATACATTTCCTCTTATACAATGTAGGTTGGAAAGCGTCCTACAAGGTTCTGAATAAATAGATAAGKGACTGAAGATTCGGGGAAAGACTTTCACGCTCTGTCTTTCTACTATCCTCAGTATTATTCATTTATTTAGAATGGTGGTTCGTTGTTGTCAAT | | | | | | |
| DenB2 | 300bp | Phage B | This study | Alexa Fluor 488 | 490/525 | FITC |
| CGTAACATCCTTCCATCAACAACAAGATACGGATTCATGGATGGAGATTCTCTTATCGAGTATAAGGGAGACATTCAGAACAAGGTTATCGACAAGTACGCTCAGTACTTCGCATCCCTTAAGTTCGAATACTTCGAAGATAAGGACTATGTAAAGAACAAGATTTTCTACGGCGGTCTTGTTGTACAGTTCAAACCATTCAGCCAGTCTGAAATTATCAAGGTTACAGTCGTTAAYAACGACGCACTCTAATAGAAAGGAGAGATTAACCTATGTTTGAAAATCTTAGAAGCCCACGTT | | | | | | |
| DenB3 | 300bp | Phage B | This study | Alexa Fluor 488 | 490/525 | FITC |
| TGATGACGGAAACATTACGATAACGATTATAAAGGAGGTAATTCATAATGAATAAYGAAACAGAAACTAAATGTGGYTTATCTTTRAACTACGATGCACTTTCAAGCGGTCTTGGAGTGCCAAAACAGCCTGAACCAGAAGTRGYTAATCCGCCGGTTAAGAGAAGAGGACGTCCTCCTAAGAAAATGGAAGA???GACYGGAATGGAAATTCAGGAGAACACTTCTGCAAACAGCAATCTCTTTTCGGTAAACACRCCATACATGGATTCATATAAAGAAACCATGACTCAGTGTGATT | | | | | | |
| DenB4 | 300bp | Phage B | This study | Alexa Fluor 488 | 490/525 | FITC |
| AACAGGTGCTTCAGTTCACTAATGATTTGACGATTCGTCTTATAAAGGTTGTGTATGGCTTAGTGCCATACACGCCTTTTTTAAGTTTTTCATAAGGAGGCTTTAAAATGGCTAAATATCTACATTATAACTTTAGTRCATGGCTTGAAGTTATAGACGATATGATAACGGAATACAACMGACATTGYAAGATTTCAAATAAGACTCTCATCGCTCTAAAGAGAGAAGTAAATAAGTTCTTTACAAATTCAGAATGTATAGGATTATTATTTACCGAAAACGATTCTCTGTTCTTCGGAA | | | | | | |
| DenB5 | 300bp | Phage B | This study | Alexa Fluor 488 | 490/525 | FITC |
| AAAGTTTACAGAACTCGAAGGCAGCGAATGGGCTACAACATACCTTCAGAATAATACTCCAAACTTTACAAAACACGGACAGCCATTACTTCAGGCAGCACTCTGTATAAATGCAGGTGCAAGAATGTTCAGTAAGCGTATCGTTGCTGATAACGCTAAGCTCGGTTCTGCAACTCTCTGCATGGGTGTTAGCGAGAAAGAATATGAAGTCGTTTCATATACAAAAGCTACTGCTGAAACAGACGGAGCTAAGAAAGTAGTTTCAACAACTCACGTTGTAGACGAAGCTACTGAAGTTGA | | | | | | |
| DenB6 | 300bp | Phage B | This study | Alexa Fluor 488 | 490/525 | FITC |
| AAGCGAACATAAGAAGGGTGAAATCGAAGTTCCTACAATCCAGCTGTCATTTACTGGWCTYTGTCTCCAGAACGATGCTATTGATGCTAAAGCTGCTGCTGCTCTCTCAATCATGAGAAATACAAACAACAACACAGCATCAAGACTCCTCGTTAACAGCTCAAGATTCGAATACAGCGAACTCTCTAAGATGCGTGCAGATGATACAGACAACAGCTACACACTCACATCAAAGGGTTCAAATATCGGTGCTGCTTCAGATGGATTCCACAAGAACACTGTTGAGTCTATGGCTAAGAA | | | | | | |
| DenB7 | 300bp | Phage B | This study | Alexa Fluor 488 | 490/525 | FITC |
| TTCGATATCAAAGCGACTGTTCATACACGAATGCAACAGCTCGAATTACTTGAATATATGAAGCGAGCATTCAATATAAAGTAYACYGAAACCAGATATGTAGACCAGGACTTCTTACTTCCTAGGAAACTAATGTATTGCATTGCTGAAGAYGAGAAGATTCCGATAGCTCCTAAYGGAGATATATTGGACCCTATGAARCTYTTATATTATATGAATAAGAATTCAAAACTCCCTATTCTRTATAAGACRAGAAGCTCTACAGGTCAGCCAGAATTCTTCTGTAGGTTTCCTGAGACT | | | | | | |
| DenC1 | 300bp | Phage C | This study | Alexa Fluor 488 | 490/525 | FITC |
| GGTGTGTTTGGTTTACCGATGTAATAAACTCCGTCTGGCAACTGAACATTTTCGTCCTGGTAATCGTTCTTTACAACTGTAACATTAATAATGTTAGAACCGCCATTATCTGCCGATTCGTTAATTATTGTATCAAAGCGATTATCAACGTCAGCATAATTATAYGAAATGTTTTCATATGTTTCTTTCAAAAGTCCATCAACTTTAATTGTTAAATCATGTTTAACACTTCCATCAACTGGACTTGTAGAAGAATATACCTCTACAACAATGTTATTTGTTGCAGAACCGTTTTCCTTA | | | | | | |
| DenC2 | 300bp | Phage C | This study | Alexa Fluor 488 | 490/525 | FITC |
| ACTTCTTGCCATTCTTTTGTAAGGAATCTTTTATCAGGAGTAAACTTTCTATCGCATTTTCCTTTTTCATAACGACTTTCTAAAAAGTGTTTTGTTCTTTTATAATTTATTTCTTTCTTTTCTTCTTCGCTGTGGCTCAGCCAAGTTTTTATTTTTTTCTCTGCTATAATCTTCTTTTCTTCTTCGCTTTTGTAAATTATGTTTTTACAATTTTCTCTTCCCCATTTTAAAAAATAACCATTTTCTTTTAAATGATTATATCTTCCTTCTTCCCAGCCTTTTGCTGTTGCTGTTCTTTCT | | | | | | |
| DenC3 | 300bp | Phage C | This study | Alexa Fluor 488 | 490/525 | FITC |
| TCTCCATCACTTTCAAAGAAAGCACGAATAAACATCTCATCTTTTCCTGAAAATGTAAATTCGTTTATATAATCCGCAAAAGTATAACTATCATTTGATTTAGTGTATGTTCCATCAGATTGTCTCCAGGCACCAGCACTATAATAGTAAAACAAGCCRCCATAATTTAAAACTAAATCTATATTATCAGAACATTTTGCAATTAATGAGTTTAAAGAGTTTGCAAAAATTTTATTGCCAGAGAAATAAAAATCTATATATGGTCTTGTAGTATCATATTTTGTGAGTTGTGCTGTTGGA | | | | | | |
| DenC4 | 300bp | Phage C | This study | Alexa Fluor 488 | 490/525 | FITC |
| CATACATTTTCTGCGTTTTCACCATAAAGAATRCCWACGTCTTCTGGTGTTMTRTTYTCATCAATYTCAAAGATATAAGGATAATCTTTATGYAAGAAATTTTTTAAAGTGTCTGCTTCAGCATGATATATTATTTCTTTTGGCCCATACAATAATTCATACCAAGCTCTTATATTTCCAGAAACAAAAAAATATGAYGGATYTYCATTCATATTTACAGTTAAATTAAAATAATGCTCAAATTCAAACATTGCTTCATAACCTTTTTCACTGAAAACATCTACCGGTATTGCAAACATT | | | | | | |
| DenC5 | 300bp | Phage C | This study | Alexa Fluor 488 | 490/525 | FITC |
| TGCATCATTTGTCTCACTTGCATAAAAAATGTTTATCATTGCTCAACTCCGATTTTTTCTCCCTTTTTTAAAAAAGGGAGGCTCTTCGATTATTTTAAAYYTATATCTGCTTTTGTCAAGCGAAGATAACTCATAATAGTTTCTGCTATTTCTCTAATTTCTTTTTGTGCATGTGGATCTACTCTAAGRTGTAAAAACTTTTTCCAGTCATCAAGAGTGCCAGTTACAACTATTGTAGAAGCAGCGCAGTTTGGTAAAATTTGTCTTGCATCTTCTGGCTTTGCACCATTTTCAATTGCT | | | | | | |
| DenC6 | 300bp | Phage C | This study | Alexa Fluor 488 | 490/525 | FITC |
| AGCAACTCCGCAAACTTTCATAACTTCATTAATAATTATTCGTTTAATTTCTACGGAATTTTTTAAATCAAGTGGTAAATTTTGATAGTCTTCTCTTGCAACATTCCATCTTTTTGCACTAATGTTGTTAAGTAATGGTCCTATATCATCTTGGTATCTTTTTTTAAGAGTCTCCATTTCATTGAGCTCTTTTTTTACCGCAGGRTTATCCTTATTTRCTTCTTTAAARTTCTTCTTAAAAGTAACGCCATCTTTACCCATTGTTTTAGCAATACTGTTAATTGCTCCAACACCACCTTT | | | | | | |
| DenC7 | 300bp | Phage C | This study | Alexa Fluor 488 | 490/525 | FITC |
| TTCCTTGAGCGCCTTTTGTTTTTCCGAATAAGTCTTCGGCAGCGTTCCCAGAAACAATTATATAAGCATTATCTCCCTTTGTTTCGGAAATTAAAACAAKYCTTCCWGCGTTATCTTTATAAATACTTGCTATGCCAATTGGGTAATTTTCTTTTATAAATTTTATAACTTCGTCTATTGTTAAATTATCTGGAAAAGTTATTTCCGTCTTTCCTTGATCTGTTTGTATTATAAGTGTTTTATCTTTTATTTGAAGCTCTTCGTCATTATGCTCTTTTTCACCAACAAGTATAGCAGGTG | | | | | | |
| DenC8 | 300bp | Phage C | This study | Alexa Fluor 488 | 490/525 | FITC |
| TTTGCTTTTGCCATTACTCCATTATCCATTAATTCGTTTGATTTTATTTGAATTATTATAGATCCAAGTTGTTGTCTAATTCTTGCAAAATCTAATAAATTACCATTCGAAGGGCTGGCAGAAAAACCATATTTAAGAGGACACCCAAAAGAAGCTAAAAAATCCTGAAACGATTTACTGCTACTTCTATGGCATTCATCAACTAATACCATTTGAAATTTTGAAACATCTAATTTTTTGACAGATTGTATTGTAGATACCATGCAAAAACCTTGCCTTACACCATTACCCGAGCAAAAT | | | | | | |
| DenD1 | 300bp | Phage D | This study | Alexa Fluor 488 | 490/525 | FITC |
| ACACAACATTTGATCTGGTGCAAATGTATCATAACCAACGGATGGAGAACGCAATGTATATCCAGAAAGACCRGAGTTTTTACGTTCACCMGCCTGYTCAGCTTTGGTCATATCYGCAATGTAATAATATCCGATTACTTCGCGATCAATACGAATTGGAATCAATTTGGTTGATGGAAGTATTCTCATATAAATACCTTTGATGTTTTGGAATTCATTAAGAATACCATCACCATTRTCATCTCCAGTATTTACCTCGGTAAACAAATCTGTTTCCTGAACGAATGGGTCTTTAAC | | | | | | |
| DenD2 | 300bp | Phage D | This study | Alexa Fluor 488 | 490/525 | FITC |
| ACAACGCATTCACAACAATCAGAAAGTGATATAAATTATATACACCCGGGAATTCCCGGGTGTATATTTTTTGTTTAACAYAGTTGGAACGTTGACTGTATGTCGAACAATCCACTACCAATGTTTGTAAACGTATGTGTTGCATACATTGGTCTGTAAATCTTGTCAATGTTGTTTCCTCTGATWGGATTATCGAATACAAACTGGAAACAATTACATGTSGTTCCRCCATATGGATAACCATTCAAAGATAAATCAATTCTAGTGTTACGTTCATCAACTCGTGCTTTGTATGTTGAA | | | | | | |
| DenD3 | 300bp | Phage D | This study | Alexa Fluor 488 | 490/525 | FITC |
| TGTTTAACACGCAAACCAAATGCGTTTATCATGAACTCACAGCTGATGTTAACTTTGTGTAAAGTTTATATCACAATGGTGTCTGCGCCATTACAACAGAAACTTTACATGAAGGGTGACAACTTGAACAAAGCAATGTTATACTTTATAACGTTCTTCTATATGCTMTTCMGAGATGTRAATGAAATAGATGTAACAACAATACCATTCAACAARGTAATGGATGATAAGATTGACAACAARGTTGCTGCATCAATCATTGAAGATGCTAAAGGCGTTCAGAACCACAACTTCATGGAT | | | | | | |
| DenD4 | 300bp | Phage D | This study | Alexa Fluor 488 | 490/525 | FITC |
| ACAATTGCAGGTGTATCATTGCAGTATGTAACAGATCTTTCCGAATCTGATAAAGCTCGCAATATCGTTCCACAGCTGATCCATGAAGATGATCCGATTTTCAAACAGATCAATCATAAYGATTCAATTGGCGTCAAGAGTAATGCAGATATGATTGAATCTTATGAGAGATGGAGAACTGAGAATCTGACAGAGACAATCACAAAACTCGAAACTGATGTATTCAAGATATTACTTGATGATTATGGTATCGATCTTTGTGTAACAAACGCRATCATTCCAATGATGGCAGCAACATAT | | | | | | |
| DenD5 | 300bp | Phage D | This study | Alexa Fluor 488 | 490/525 | FITC |
| AACACCGGATACAGAAACAACATCAGAATCARCACCGATGGAGAAACTCGATCCACCAACATTYGAACCCCATRTTCCAGCTCCATACCCTATAGAACCAAACATTTCAACAGAGGTCAAAACTGATGAAATCGATTATGGTGATGATGATCTGGAAAATGAACTCAAAGCTGAGGACGATGCACGTCTCAAAGAACGTGAAGCAATGTATGCCAATGCAAAGAAAAACGCAMTCCCYGATGCATTCCAGGCTCCTGATGAAAAWGATGAAAATTATAATCGTGAAGCCATTGGATTTCA | | | | | | |
| DenD6 | 300bp | Phage D | This study | Alexa Fluor 488 | 490/525 | FITC |
| TGTCGAAACCCGTTTGAGATTATTACAAGGATTAATGGATACAGATGGATCAATATCGTATTGTGGTGGTAGATATCACACGACGTATTCATCAACATCWAARAARYTACTTGAACAGATTCAATGGATCGTTCGTTCTTTGGGTTATTCGGCGAATATCATTGTTGATAAACGCAAAGACAAATATACATCTGGTTTCTGTGGTACGTTGATATTCAGAGTACCAAACACATATAAACGAAATTTGTTCACATTGTCAAATAAGCTTGAACGTGCGATGCAGGCAACTGATAAAGAACA | | | | | | |
| DenE1 | 300bp | Phage E | This study | Alexa Fluor 488 | 490/525 | FITC |
| TGCAGTTTCAAGTTGGAGCAAATGCAGACCAGAATATGAGAATTTACATTGGAACTATGACTGCTGAAGCACTTGGTTTAAGAAACTCTCAAGGAGAGGAAAATATAATTTCGGTAGAAGATCCAGAAAGCTCTAATGCTGTAATAGCAACAATAGACAATGCTCTTCAAAATGTTTCAAAACAGAGGGCTGATTTGGGTGCTTATCAAAACAGAATGGAAACAATGCAGAAAGGTATAGATGTTGCATCAGAAAATCTTACAGCAGCTAATTCAAGAATCGCTGATACAGAAATGGCAA | | | | | | |
| DenE2 | 300bp | Phage E | This study | Alexa Fluor 488 | 490/525 | FITC |
| TGGCTGTTTGAATAATTCCTGCAGCCATTTCATGCATTGCTACAAACAAAGCACGTGTAACAGTTGTATCAATATCATTATATAAATATATATGATTTTTTACAGAAATTATAGGTGAGCTTGGTTGTTCTAATGGATTTTGGATGGCCGTTAAATCTTCATCAYAATTAATTTCTTCTTTCATTTTTTCTCCTATTTAATTTTTCTTTTATTATATCAAATTTTTTTATTTGGTCAAGTTAATCATATAACATTACGCCGAATAATAAAAGAAGAATTAACACCCGAACAAGCAGAAAA | | | | | | |
| DenE3 | 300bp | Phage E | This study | Alexa Fluor 488 | 490/525 | FITC |
| TCGAATGTCCTGTTTTTACAGCAGCTCAAATCAATAGAAACGGAATGGGCGATAAAGGCGGAACAAAGGGAGTAGTAACAGCAAAGGATTTGTCGGAATCAAGAGCAATTCTTGATACAGCTGATTATTTGTTAATTATTAATCAAACTGATTCAGAGAAAAAATTAGGCGAAAAAGATCATATAGCAGAACAGAGAATTTATATTGATAAAAACAGAAATGGTACATCTGGTGATACTTTAACATTTACGATTGATTATAATACAATGAGCATTATTGAAGGAAAGAAAAAGCGTGGAT | | | | | | |
| DenE4 | 300bp | Phage E | This study | Alexa Fluor 488 | 490/525 | FITC |
| AACATATTGCAGCAGTACAGCTTAGAATGGAAAGATTAGCCATAGAGCTATCAGATAGAAGTCAAAAACATGATGAATCAAAATTAGAAAGTCCAGAACATTCTATGTGGCTCTGTATGGACAAAGATTCATTAAAACCCAAATATGGCACACCAGAATATTTTCAAAAAATGAAAAAATTTAAATATGTTTTTGACCAGCACTACAAAGATCCAAGAAACAGGCATCATCCAGAGCATTGGGAAAATGGTATTTATGATATGAATCTTATAGATATTACAGAAATGTTGTGCGATTGGA | | | | | | |
| DenE5 | 300bp | Phage E | This study | Alexa Fluor 488 | 490/525 | FITC |
| CGTACTTTTCAAAAGCAACACGTGGAATAGGATCACCATTCTTGTTAGTGTGACCTTTGTACTTTCCTTCTACTACAACTTTAAAAGTATCTCCACCATTAACCTGTGCTGTTTTAACAACTACACTTTCTGCTCCAAATCCAGGTGCTACTACATCAATGATAAGTCTCTCATCTGTAACTGACTTGTAAAAATTTTTCATATTTAACTCCTTATTTATTATAAATTATTTTTATTTTTTTGTCAATAATTATTCTTCTGTTCCTGTGCTATCAGCATCGGCATTTTCTACTGCTTCAA | | | | | | |
| DenE6 | 300bp | Phage E | This study | Alexa Fluor 488 | 490/525 | FITC |
| TTCGTTTCCACAACAATCGCATACAAATGTTTTATCTTTTGTACAATCATAGCATAAAAAACTTTCATTATTTATAACAAATCCTGCTTTATCTTCATGGCAAGAAGAACATTTACAAGTTTTAAACAACATATCAACATGAAGTCCAAGTTTTTTCCATTTTGGAATGTTCCAACCTGGACTATTAAAGCCTAAACCATTAGTATTTATATTACTAAGTTTAAAAGAAGAATAATTATATTTATTACAATTCAACAAATTTTTTGTGAAGAATTGTTGACCATCTTTGCCATTTCCAA | | | | | | |
| DenE7 | 300bp | Phage E | This study | Alexa Fluor 488 | 490/525 | FITC |
| AAACTTTAATTTGCCAGCCTGCTGTTTTATTTAATCCTAACCAAATAGCGGCTTCTCTTAAACTATTAAAAACAACATTAGATTCTAAGCATTTTATTTTAATACCTTTAAATTGTTTTATCTTTTCCTTTACTTTCTTTGACAAATGCTTTCCTTTCATATGAGAAGTTCTTTCGGTTAAAATAGGGTTATAGCCGTTTATTTTCCAATTTTTGCGTTAGTTAATTTCTTACGATTGACTTCTTCTAATATAAGCTCATTTTTATGTTGCTTAAATTCTTTTGCCTCATTTGCCCAA | | | | | | |
| EUB338 | 18bp | Bacteria | Amann 1990 | Cy3 | 554/568 | H orange |
| GCT GCC TCC CGT AGG AGT | | | | | | |
| nonEUB338 | 18bp | 0 | Amann 1990 | Cy5 | 649/666 | Cy5 |
| ACT CCT ACG GGA GGC AGC | | | | | | |
| *^a^Probe sequences listed 5’- to -3’.* | | | | | | |

## S3 Table: Primer sequences

| All primers | | | | |
| --- | --- | --- | --- | --- |
| Primer name |  | Sequence |  | Product size |
| A1F | 5’- | AGCATTTACTGAAAACTCAAACGGT | -3’ | 300bp |
| A1R | 5’- | TCAAATGACTGCTTTTCACCGTT | -3’ | 300bp |
| A2F | 5’- | AAGGTGTTGGACTTGTAACTGAGAT | -3’ | 300bp |
| A2R | 5’- | TGCCAAGTTCTTTTGCGATACAT | -3’ | 300bp |
| A3F | 5’- | GGGAGATTACCTTTCACAAGCAAAA | -3’ | 300bp |
| A3R | 5’- | AGATATGTTGAAACGATTCCCGAA | -3’ | 300bp |
| A4F | 5’- | TGCTTGGGTTGGAGCAAAATATT | -3’ | 300bp |
| A4R | 5’- | TCTTCGCAGTTGTAAATTGTTCCT | -3’ | 300bp |
| *A5F | 5’- | ATTGCAACAAATTGACTTACCGC | -3’ | 300bp |
| *A5R | 5’- | GCTTCTGAGAAAACCCAAACATT | -3’ | 300bp |
| *A6F | 5’- | TCACTTCCACCATTTGACATCAA | -3’ | 300bp |
| *A6R | 5’- | TGACAAAGCAGCAGGATTTTTAG | -3’ | 300bp |
| B1F | 5’- | TTATTACGGTCATTTTCTCATCTGCC | -3’ | 300bp |
| B1R | 5’- | ATTGACAACAACGAACCACCATT | -3’ | 300bp |
| B2F | 5’- | CGTAACATCCTTCCATCAACAACAA | -3’ | 300bp |
| B2R | 5’- | AACGTGGGCTTCTAAGATTTTCA | -3’ | 300bp |
| *B3F | 5’- | TGATGACGGAAACATTACGATAACG | -3’ | 300bp |
| *B3R | 5’- | AATCACACTGAGTCATGGTTTCT | -3’ | 300bp |
| *B4F | 5’- | AACAGGTGCTTCAGTTCACTAAT | -3’ | 300bp |
| *B4R | 5’- | TTCCGAAGAACAGAGAATCGTTT | -3’ | 300bp |
| B5F | 5’- | AAAGTTTACAGAACTCGAAGGCA | -3’ | 300bp |
| B5R | 5’- | TCAACTTCAGTAGCTTCGTCTACAA | -3’ | 300bp |
| B6F | 5’- | AAGCGAACATAAGAAGGGTGAAA | -3’ | 300bp |
| B6R | 5’- | TTCTTAGCCATAGACTCAACAGTGT | -3’ | 300bp |
| *B7F | 5’- | TTCGATATCAAAGCGACTGTTCA | -3’ | 300bp |
| *B7R | 5’- | AGTCTCAGGAAACCTACAGAAGAAT | -3’ | 300bp |
| C1F | 5’- | GGTGTGTTTGGTTTACCGATGTAAT | -3’ | 300bp |
| C1R | 5’- | TAAGGAAAACGGTTCTGCAACAA | -3’ | 300bp |
| C2F | 5’- | ACTTCTTGCCATTCTTTTGTAAGGA | -3’ | 300bp |
| C2R | 5’- | AGAAAGAACAGCAACAGCAAAAG | -3’ | 300bp |
| C3F | 5’- | TCTCCATCACTTTCAAAGAAAGCA | -3’ | 300bp |
| C3R | 5’- | TCCAACAGCACAACTCACAAAAT | -3’ | 300bp |
| C4F | 5’- | CATACATTTTCTGCGTTTTCACCA | -3’ | 300bp |
| C4R | 5’- | AATGTTTGCAATACCGGTAGATGT | -3’ | 300bp |
| *C5F | 5’- | TGCATCATTTGTCTCACTTGCAT | -3’ | 300bp |
| *C5R | 5’- | AGCAATTGAAAATGGTGCAAAGC | -3’ | 300bp |
| C6F | 5’- | AGCAACTCCGCAAACTTTCATAA | -3’ | 300bp |
| C6R | 5’- | AAAGGTGGTGTTGGAGCAATTAA | -3’ | 300bp |
| C7F | 5’- | TTCCTTGAGCGCCTTTTGTTTTT | -3’ | 300bp |
| C7R | 5’- | CACCTGCTATACTTGTTGGTGAAAA | -3’ | 300bp |
| C8F | 5’- | TTTGCTTTTGCCATTACTCCATT | -3’ | 300bp |
| C8R | 5’- | ATTTTGCTCGGGTAATGGTGTAA | -3’ | 300bp |
| D1F | 5’- | AATGTTAAAGACCCATTCGTTCAGG | -3’ | 300bp |
| D1R | 5’- | ACACAACATTTGATCTGGTGCAA | -3’ | 300bp |
| D2F | 5’- | ACAACGCATTCACAACAATCAGA | -3’ | 300bp |
| D2R | 5’- | TTCAACATACAAAGCACGAGTTGA | -3’ | 300bp |
| *D3F | 5’- | TGTTTAACACGCAAACCAAATGC | -3’ | 300bp |
| *D3R | 5’- | ATCCATGAAGTTGTGGTTCTGAA | -3’ | 300bp |
| D4F | 5’- | ACAATTGCAGGTGTATCATTGCA | -3’ | 300bp |
| D4R | 5’- | ATATGTTGCTGCCATCATTGGAAT | -3’ | 300bp |
| D5F | 5’- | AACACCGGATACAGAAACAACAT | -3’ | 300bp |
| D5R | 5’- | TGAAATCCAATGGCTTCACGATT | -3’ | 300bp |
| *D6F | 5’- | TGTCGAAACCCGTTTGAGATTAT | -3’ | 300bp |
| *D6R | 5’- | TGTTCTTTATCAGTTGCCTGCAT | -3’ | 300bp |
| *E1F | 5’- | TGCAGTTTCAAGTTGGAGCAAAT | -3’ | 300bp |
| *E1R | 5’- | TTGCCATTTCTGTATCAGCGATT | -3’ | 300bp |
| *E2F | 5’- | TGGCTGTTTGAATAATTCCTGCA | -3’ | 300bp |
| *E2R | 5’- | TTTTCTGCTTGTTCGGGTGTTAA | -3’ | 300bp |
| *E3F | 5’- | TCGAATGTCCTGTTTTTACAGCA | -3’ | 300bp |
| *E3R | 5’- | ATCCACGCTTTTTCTTTCCTTCA | -3’ | 300bp |
| *E4F | 5’- | AACATATTGCAGCAGTACAGCTT | -3’ | 300bp |
| *E4R | 5’- | TCCAATCGCACAACATTTCTGTA | -3’ | 300bp |
| *E5F | 5’- | CGTACTTTTCAAAAGCAACACGT | -3’ | 300bp |
| *E5R | 5’- | TTGAAGCAGTAGAAAATGCCGAT | -3’ | 300bp |
| *E6F | 5’- | TTCGTTTCCACAACAATCGCATA | -3’ | 300bp |
| *E6R | 5’- | TTGGAAATGGCAAAGATGGTCAA | -3’ | 300bp |
| *E7F | 5’- | AAACTTTAATTTGCCAGCCTGCT | -3’ | 300bp |
| *E7R | 5’- | TTGGGCAAATGAGGCAAAAGAAT | -3’ | 300bp |
| T4_1F | 5’- | ATGGCTCACTTTAATGAATG | -3’ |  |
| T4_1R | 5’- | TATCCATTATCTTGACGTTC | -3’ |  |
| T4_2F | 5’- | TGGATATTAAACAAAAATTTTATAG | -3’ |  |
| T4_2R | 5’- | CGCGAGTACTAGCCATG | -3’ |  |
| **Successful primers used for probe synthesis* | | | | |

## S4 Table: PhageFISH buffer components

| Permeabilisation buffer | 1x PBS pH 7.4, 0.1M Tris-HCl pH 8.0, 0.05M EDTA, 0.5mg/ml lysozyme |
| --- | --- |
| Hybridisation buffer I | 50% formamide (v/v), 10% (w/v) dextran sulphate, 0.9M NaCl, 0.02M Tris-HCl pH 8.0, 1% (w/v) nucleic acid blocking solution, 0.25mg/ml salmon sperm DNA, 0.25mg/ml yeast RNA, 0.02% (w/v) SDS, and 1ng/µl cyanine-labelled DNA probe |
| Hybridisation buffer II | 35% formamide (v/v), 10% (w/v) dextran sulphate, 5x SSC, 20mM EDTA, 1% (w/v) nucleic acid blocking solution, 0.35mg/ml salmon sperm DNA, 0.25mg/ml yeast RNA, and 0.1% (w/v) SDS |
| Wash buffer I | 28mM NaCl, 5mM EDTA, 20mM Tris-HCl pH 8.0, 0.01% (w/v) SDS |
| Wash buffer II | 2xSSC, 0.1% (w/v) SDS |
| Wash buffer III | 0.1xSSC, 0.1% (w/v) SDS |
| CARD buffer | 20% (w/v) dextran sulphate, 0.1% (v/v) nucleic acid blocking reagent, 2M NaCl, 0.0015% H_2_O_2_, Alexa tyramides at concentration recommended by supplier |
|  | |

## S5 Table: Phage detection across faecal samples

| _↓ DNA_  ^→ Primer^ | | A | | | | | | B | | | | | | | C | | | | | | | | D | | | | | | E | | | | | | |
| --- | --- | --- | --- | --- | --- | --- | --- | --- | --- | --- | --- | --- | --- | --- | --- | --- | --- | --- | --- | --- | --- | --- | --- | --- | --- | --- | --- | --- | --- | --- | --- | --- | --- | --- | --- |
|  |  | 1 | 2 | 3 | 4 | 5 | 6 | 1 | 2 | 3 | 4 | 5 | 6 | 7 | 1 | 2 | 3 | 4 | 5 | 6 | 7 | 8 | 1 | 2 | 3 | 4 | 5 | 6 | 1 | 2 | 3 | 4 | 5 | 6 | 7 |
| A | F12 | - | - | - | - | + | + | - | - | - | - | - | - | - | - | - | + | - | + | - | - | + | - | - | - | - | - | - | - | - | - | - | - | - | - |
|  | F33 | - | - | - | - | + | + | - | - | + | - | + | - | - | ND | ND | + | ND | + | ND | + | ND | - | - | + | - | - | - | - | - | - | - | - | - | - |
| B | F42 | - | - | - | - | + | + | - | - | + | + | - | - | + | ND | ND | + | ND | + | ND | + | ND | - | - | + | - | - | - | - | - | - | - | - | - | - |
|  | F71 | - | - | - | - | + | + | - | - | + | + | - | - | + | - | - | + | - | + | - | - | - | - | - | + | - | - | + | - | - | - | - | - | - | - |
| C | F67 | - | - | - | - | - | - | - | - | + | - | + | - | - | - | - | - | - | + | - | - | - | - | - | - | - | - | - | - | - | - | - | - | - | - |
|  | F78 | - | - | - | - | + | + | - | - | - | - | - | - | - | - | - | + | - | + | - | - | - | - | - | - | - | - | - | - | - | - | - | - | - | - |
| D | F95 | - | - | - | - | + | + | - | - | + | - | - | - | - | ND | ND | - | ND | - | ND | - | ND | - | - | + | - | - | + | - | - | - | - | - | - | - |
|  | F101 | - | - | - | - | + | + | - | - | + | + | - | - | + | - | - | - | - | - | - | - | - | - | - | + | - | - | + | + | + | + | + | + | + | - |
| E | F49 | - | - | - | - | + | + | - | - | - | - | - | - | - | - | - | - | - | - | - | - | - | - | - | - | - | - | - | + | + | + | + | + | + | + |
|  | F59 | - | - | - | - | - | - | ND | - | - | - | ND | ND | - | ND | ND | - | ND | - | ND | - | ND | ND | - | - | ND | - | ND | + | + | + | + | + | + | + |
| *^+^Single band at 300bp visible in gel*  *^-^No band, smudged bands, or inconclusive data*  *^ND^No data* | | | | | | | | | | | | | | | | | | | | | | | | | | | | | | | | | | | |

| *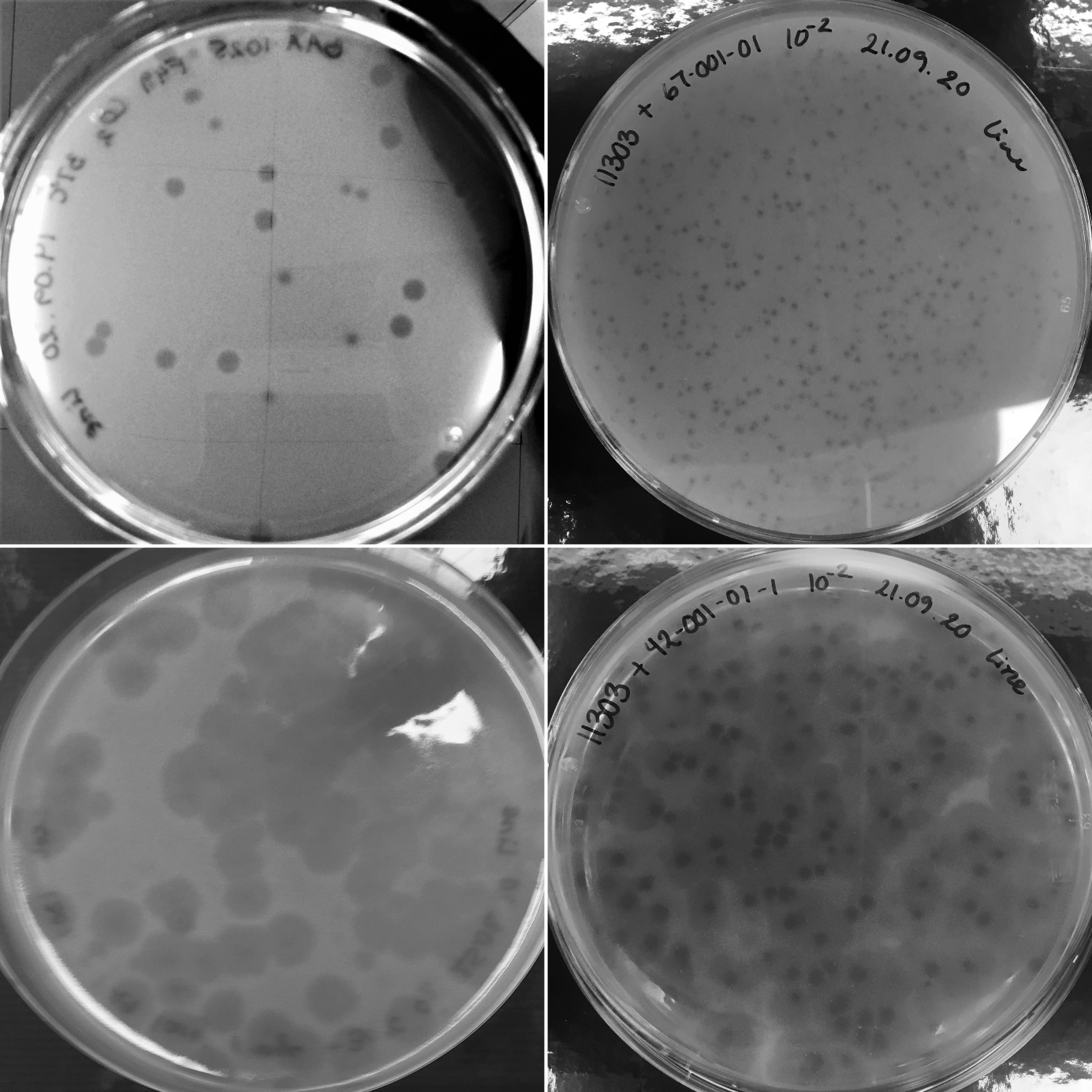* |
| --- |
| *Figure S1: Faecal viral population plated on Escherichia coli in LB 0.35% agar. Different plaque morphologies dominate phage populations.* |

# **SUPPLEMENTARY FIGURES**

## S1 Figure: Plaque morphology

## S2 Figure: Background fluorescence in FITC spectrum

| 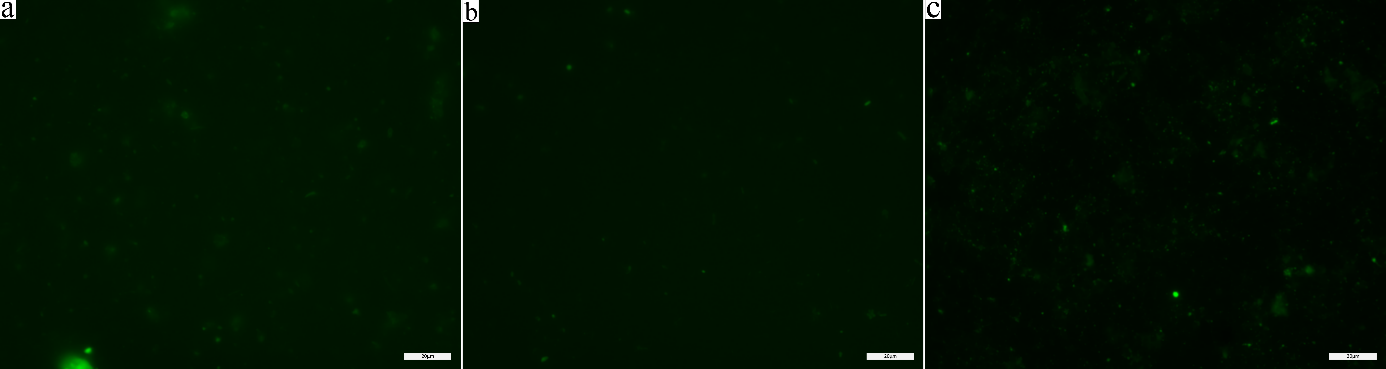 |
| --- |
| *Figure S2: Debris in faecal samples is visible in the FITC filter spectrum (green) but is distinguishable from phage signals. a) Smear of faecal sample F12 stained with DAPI dye viewed in the FITC filter. Auto-fluorescent debris is vaguely visible and some more intense signals are visible as large dots. No DNA probes are hybridised to this sample. b) Faecal sample F12 stained with DAPI dye, EUB338-Cy3, nonEUB338-Cy5, and probe group E amplified with Alexa Fluor 488 tyramides viewed through FITC filter. The group E probe sequences are not found in the F12 metagenome and only debris is visible. c) Faecal sample F49 stained with DAPI dye, EUB338-Cy3, nonEUB338-Cy5, and probe group E amplified with Alexa Fluor 488 tyramides viewed through FITC filter. Phage group E probe sequences are present in the F49 metagenome. Phage signals are clearly visible as single dots of intense signal and intense signals in the shape of single cells.* |

## S3 Figure: PhageFISH signals from all phages

| *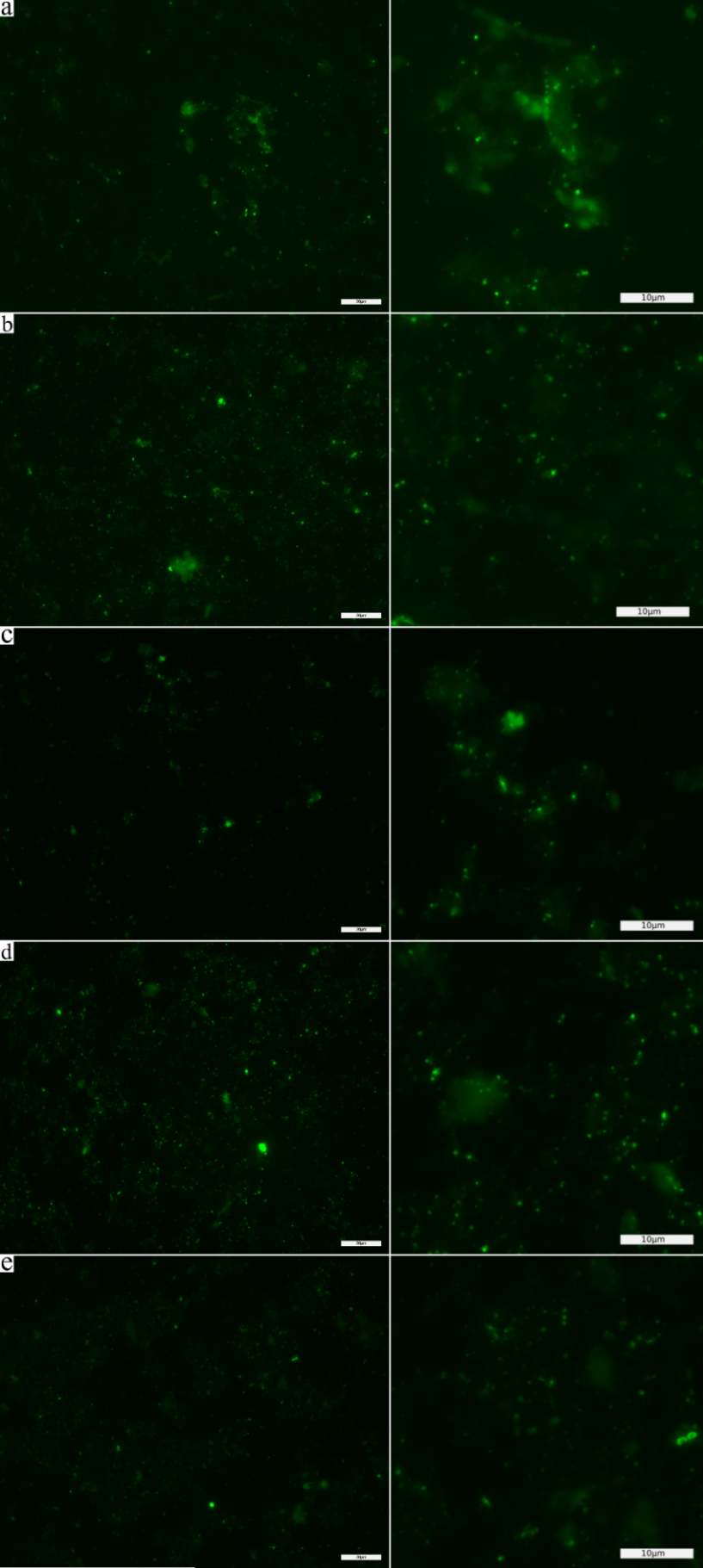*  *Figure S3: Each probe group produces signals when hybridised to target faecal samples. There is no observable difference between signal intensity and abundance when using one or several probes. a) Smear of faecal sample F12 probed with phage group A, b) Smear of faecal sample F42 probed with phage group B, c) Smear of faecal sample F78 probed with phage group C, d) Smear of faecal sample F95 probed with phage group D, e) Smear of faecal sample F49 probed with phage group E.* |
| --- |

## S4 Figure: PhageFISH micrographs for each faecal sample

| 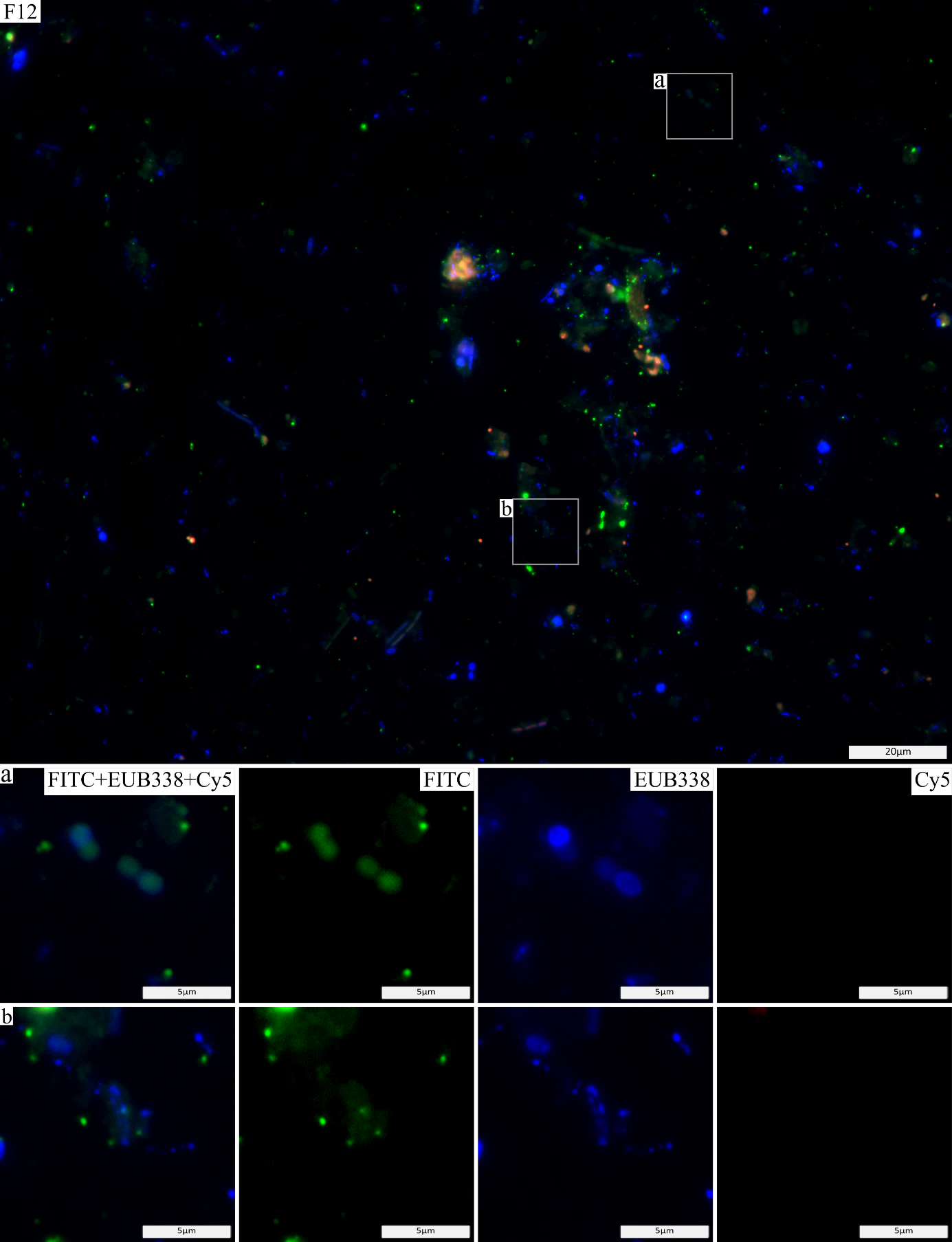 | |
| --- | --- |
|  | |
| 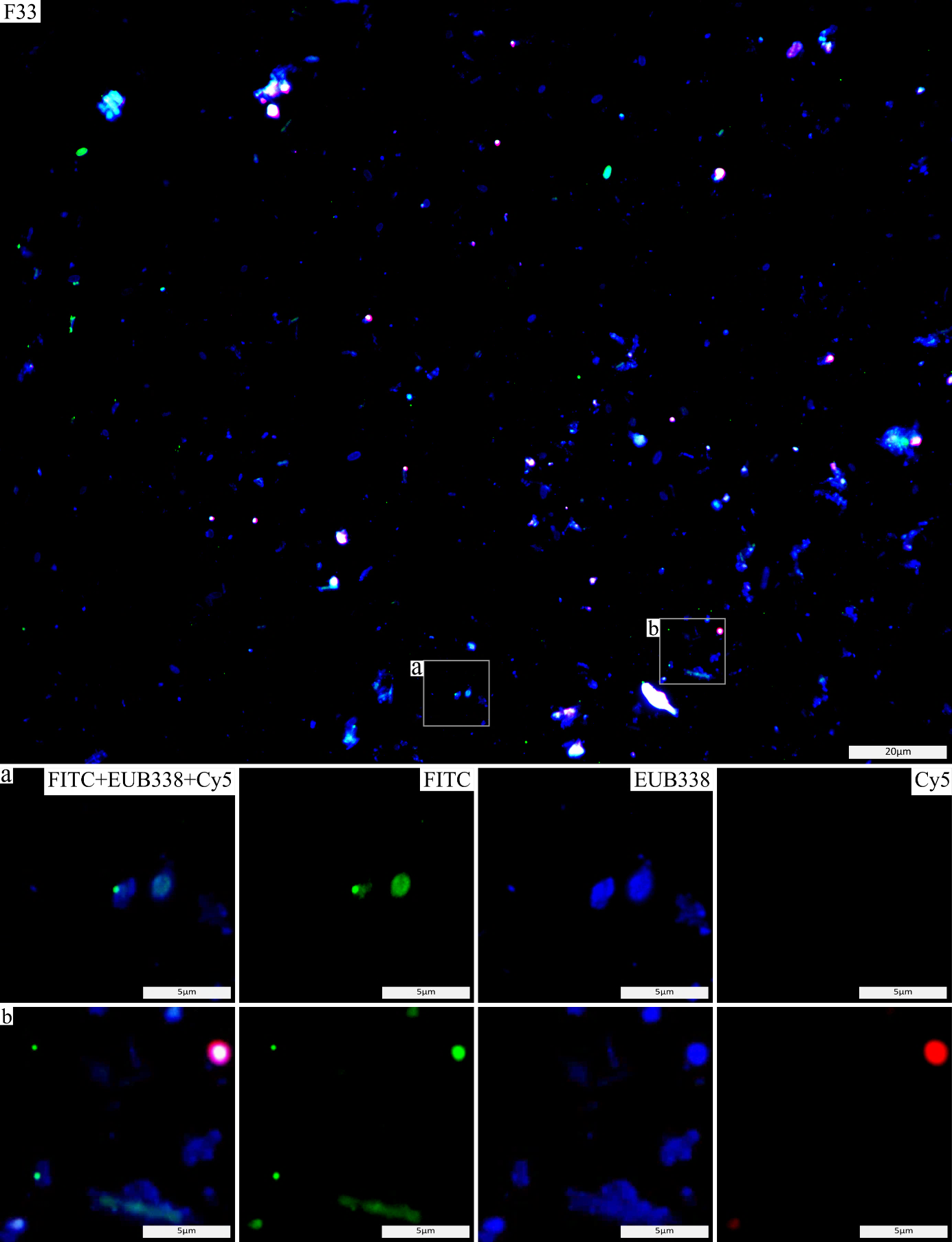 |  |

| 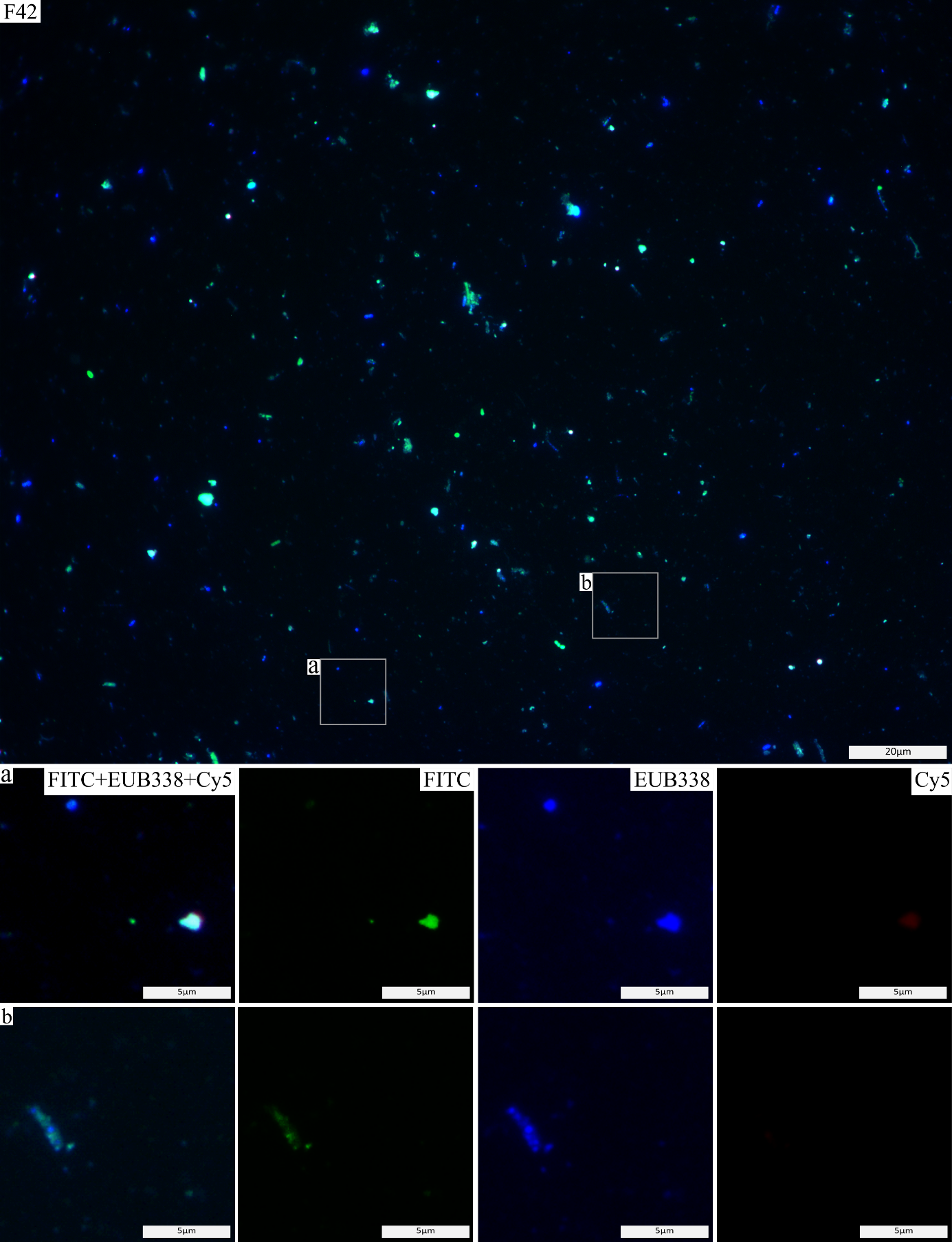 |
| --- |
|  |

| 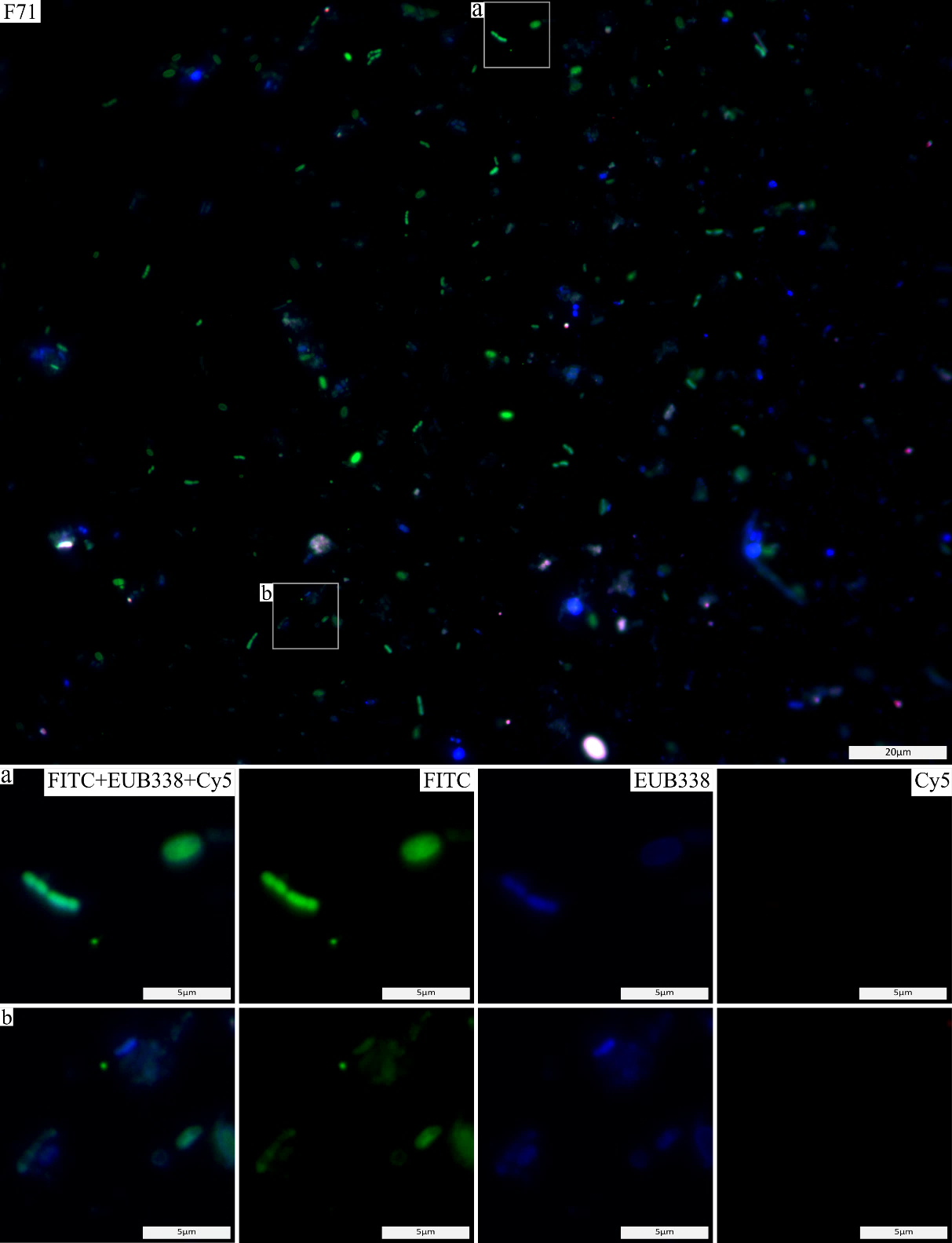 |
| --- |
|  |

| 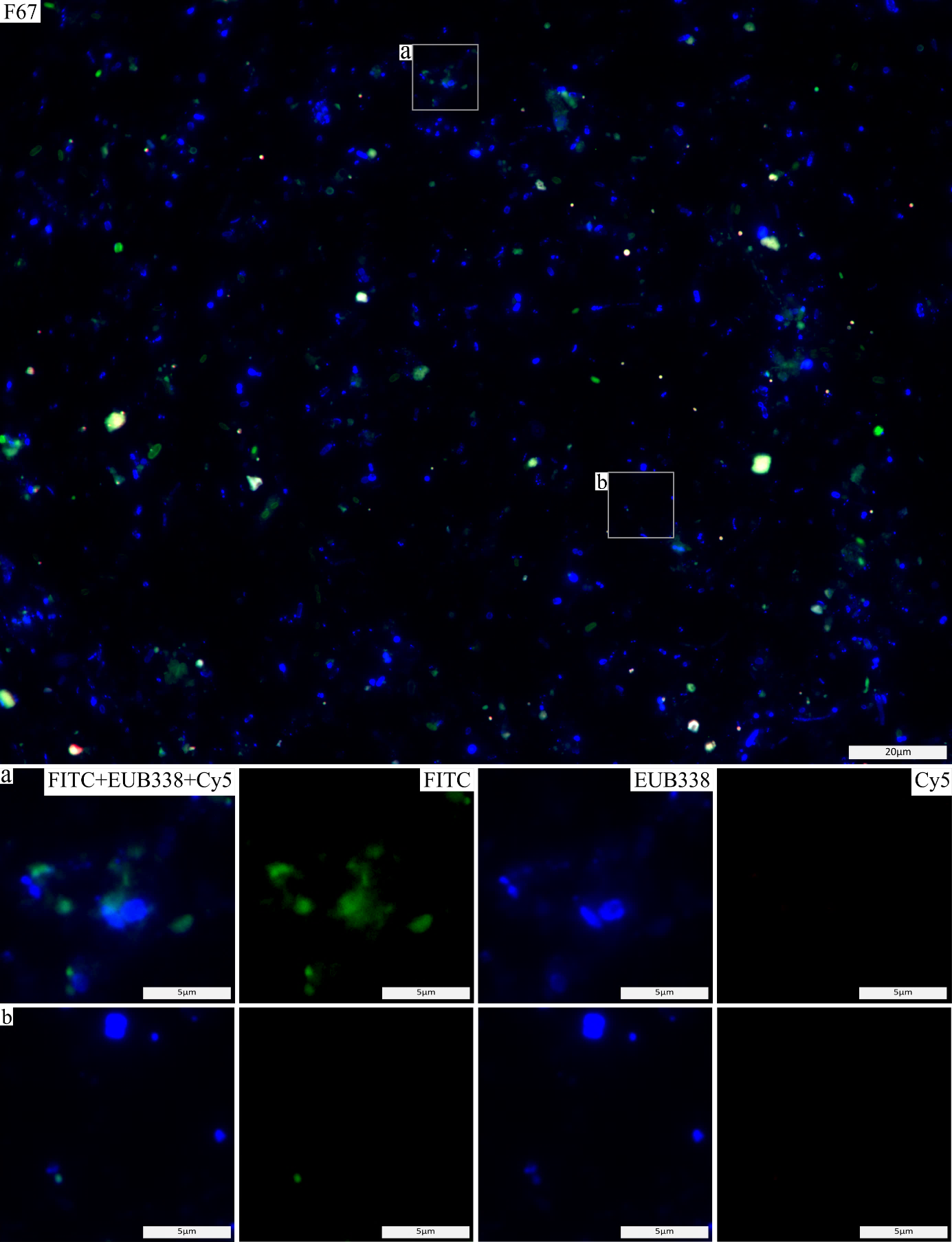 |
| --- |
|  |

| 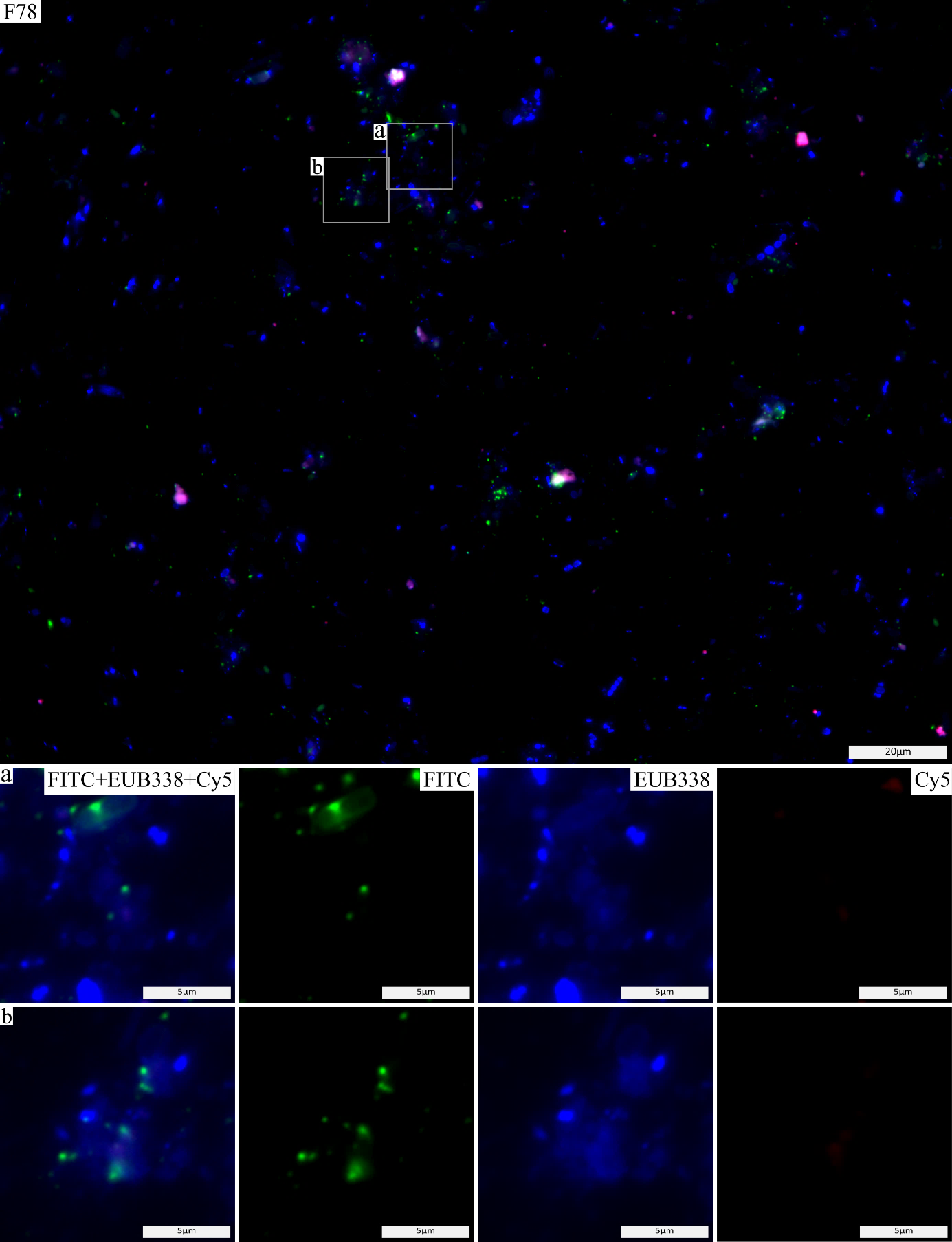 |
| --- |
|  |

| 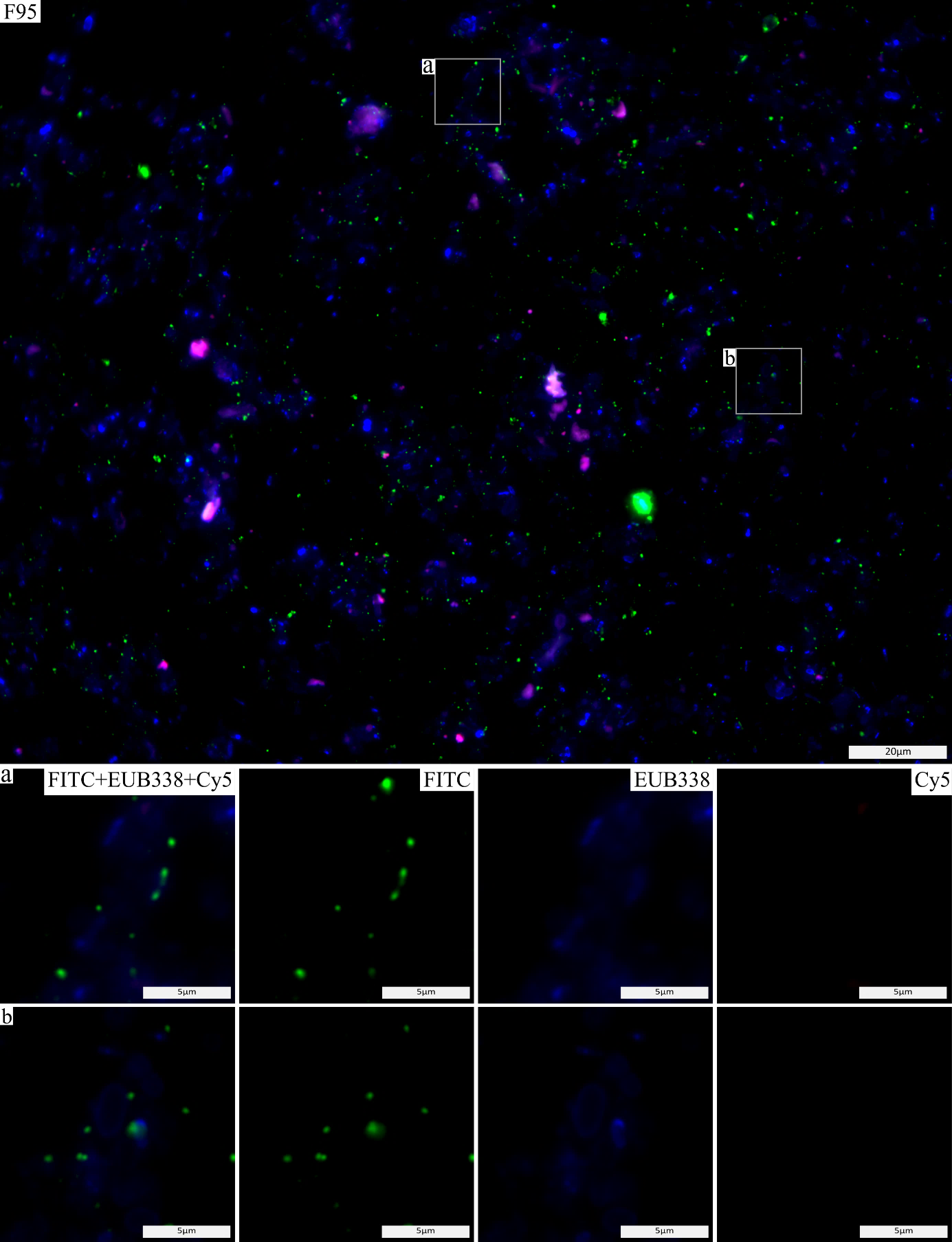 |
| --- |
|  |

| 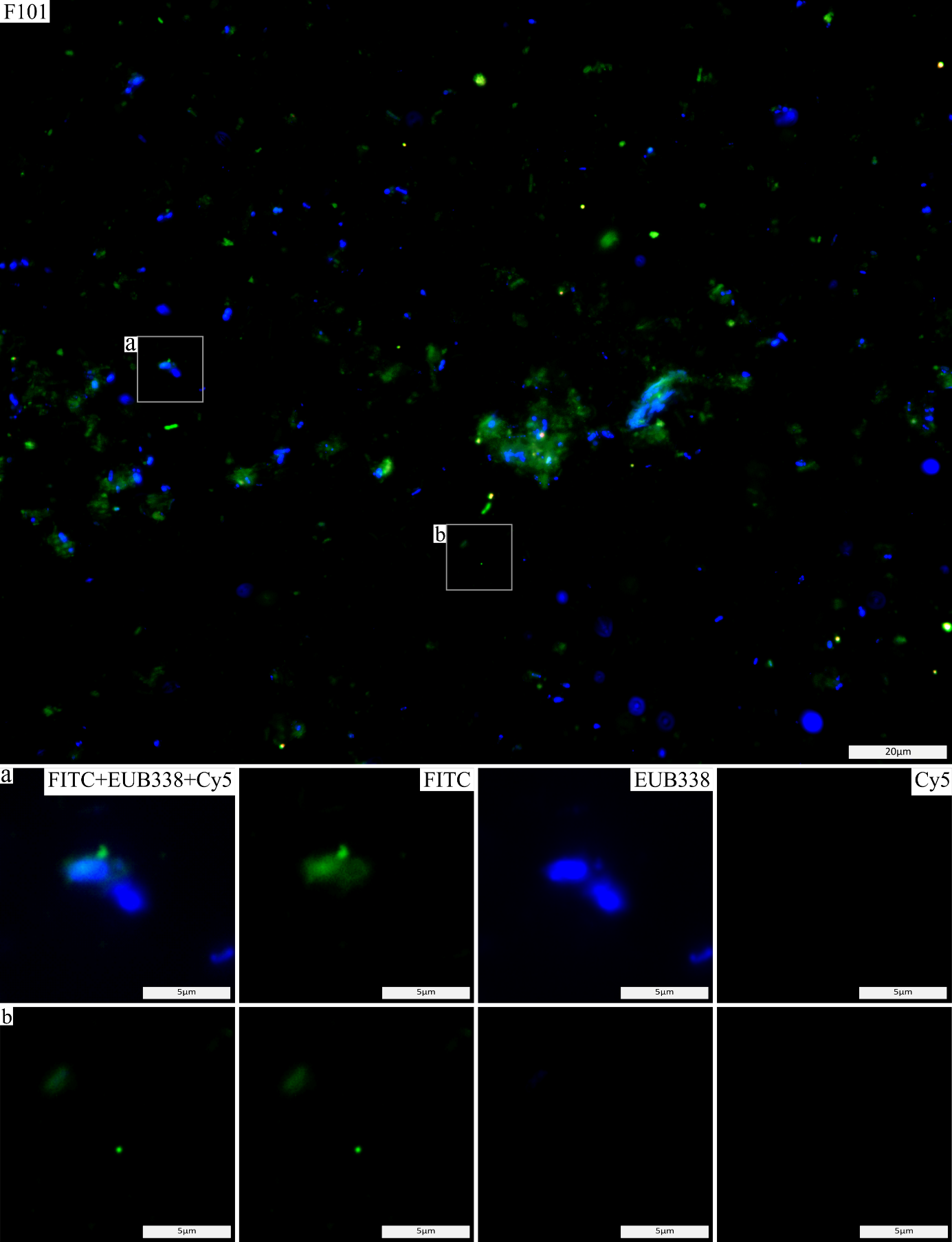 |
| --- |
|  |

| 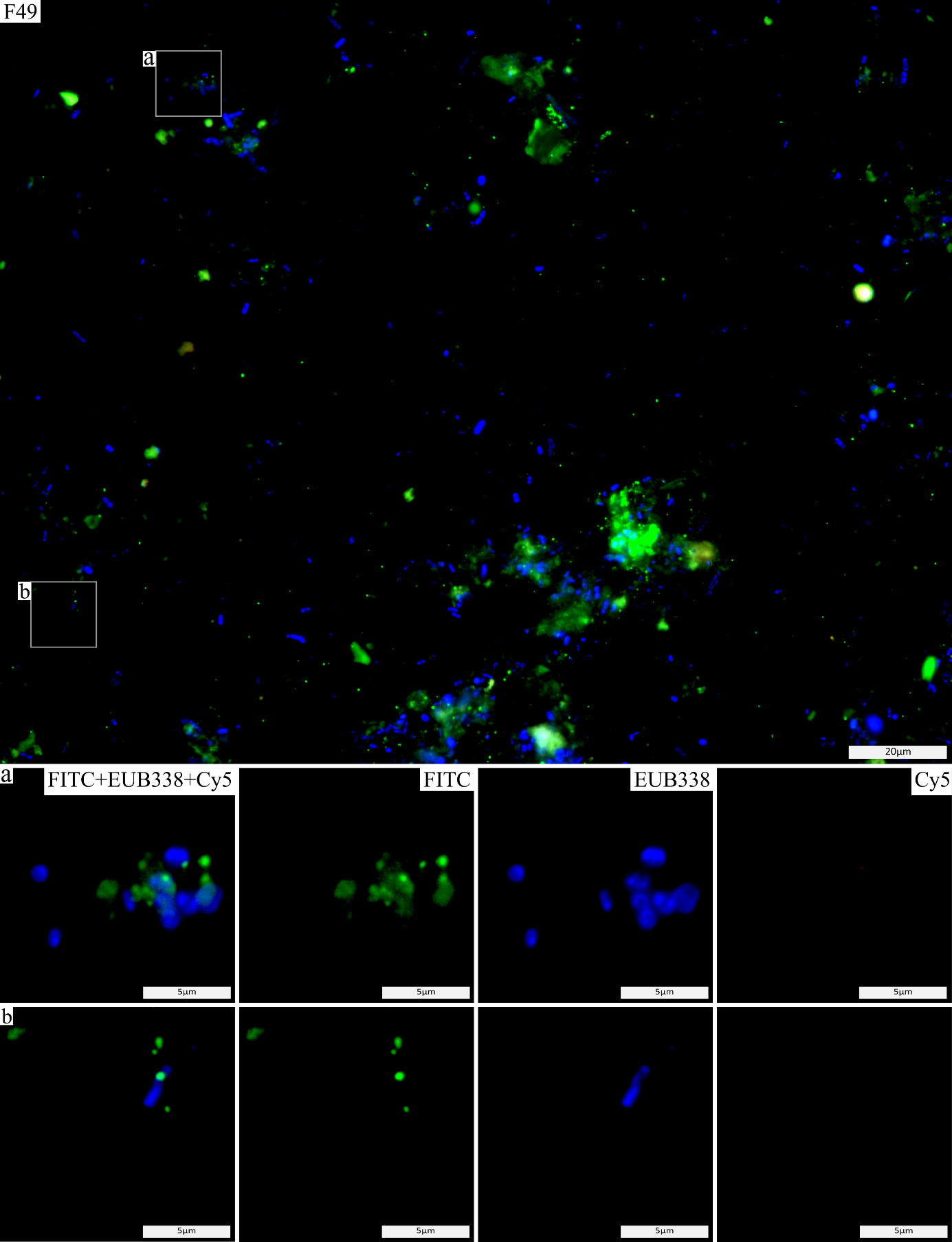 |
| --- |
|  |

| 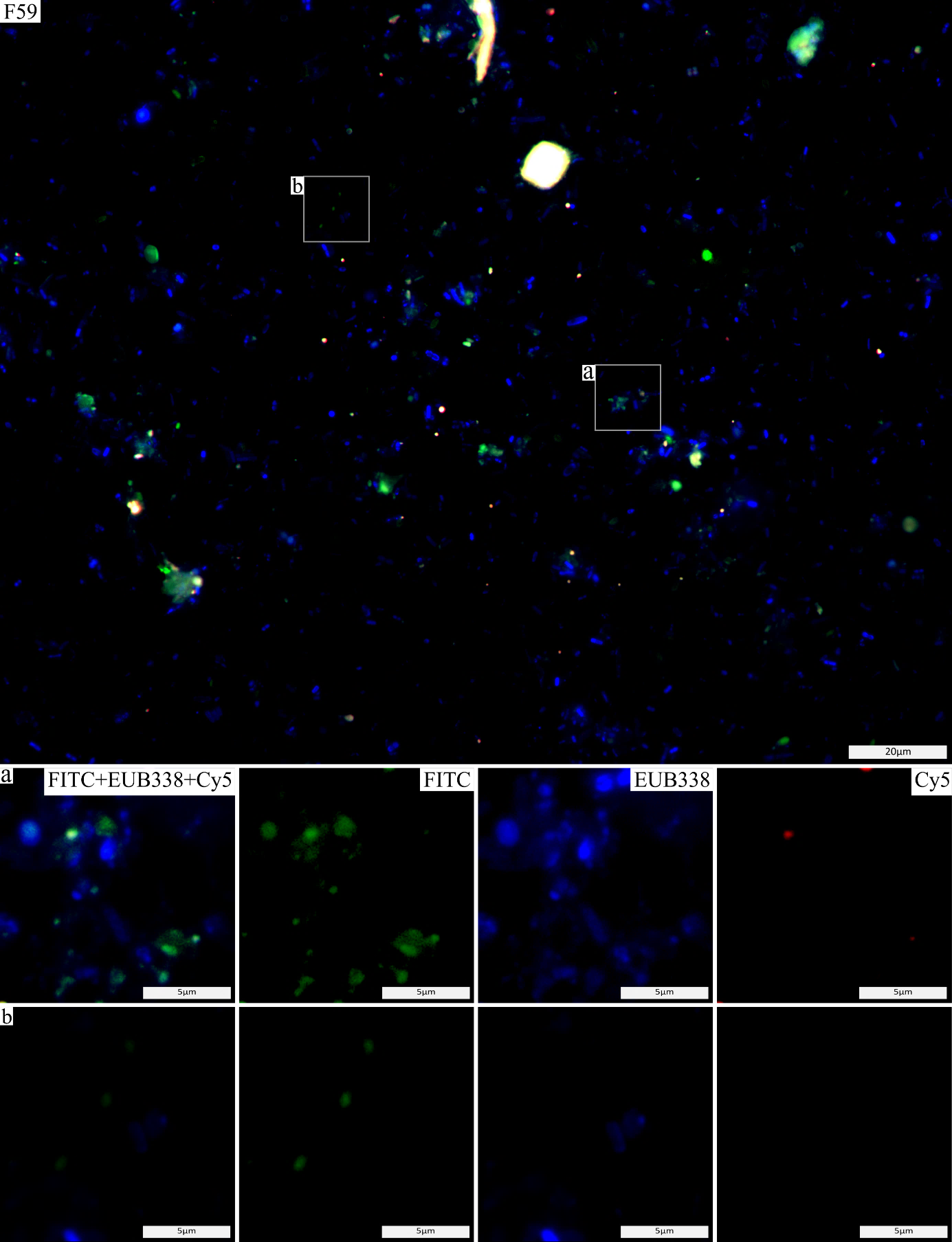 |
| --- |
| *Figure S4: Smear of faecal samples F12, F33, F42, F71, F67, F78, F95, F101, F49, F59 respectively hybridised with universal bacterial probe EUB338-Cy3 (blue), non-specific probe nonEUB338-Cy5 (red), and phage probe group A (F12, F33), B (F42, F71), C (F67, F78), D (F95, F101), or E (F49, F59) amplified with AlexaFluor 488 tyramides (green). Scale bars indicate 20µm and 5µm. Free phages (cyan arrow), early stage infection (yellow arrow), advanced infection (red arrow), and burst (magenta arrow) indicate phage behaviour observed in each sample.* |

## S5 Figure: nonEUB338-Cy5 as negative control for background fluorescence

| **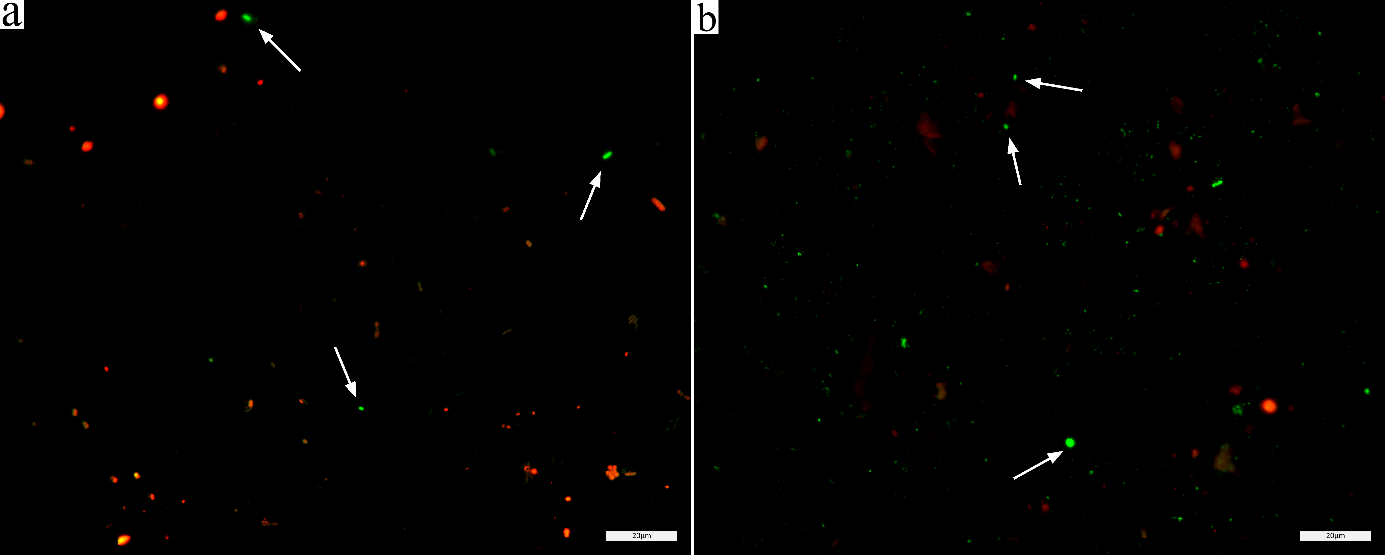** |
| --- |
| *Figure S5: nonEUB338-Cy5 works as a negative control for background fluorescence a) Smear of faecal sample F12 probed with EUB338-Cy3 (not shown), nonEUB338-Cy5 (red), and non-target probe group E (green). All background fluorescence co-localises with negative control signals except a few highly intense Bacillus-shaped signals (white arrows). b) Smear of faecal sample F49 probed with on-target probe group E (green), negative control nonEUB338-Cy5 (red), and EUB338-Cy3 (not shown). Single intense signal are clearly visible and distinguishable compared to panel a. The intense Bacillus-like signals are recognisable from the remaining signals and should be disregarded (white arrows).* |

## S6 Figure: nonEUB338-Cy5 as negative control

| 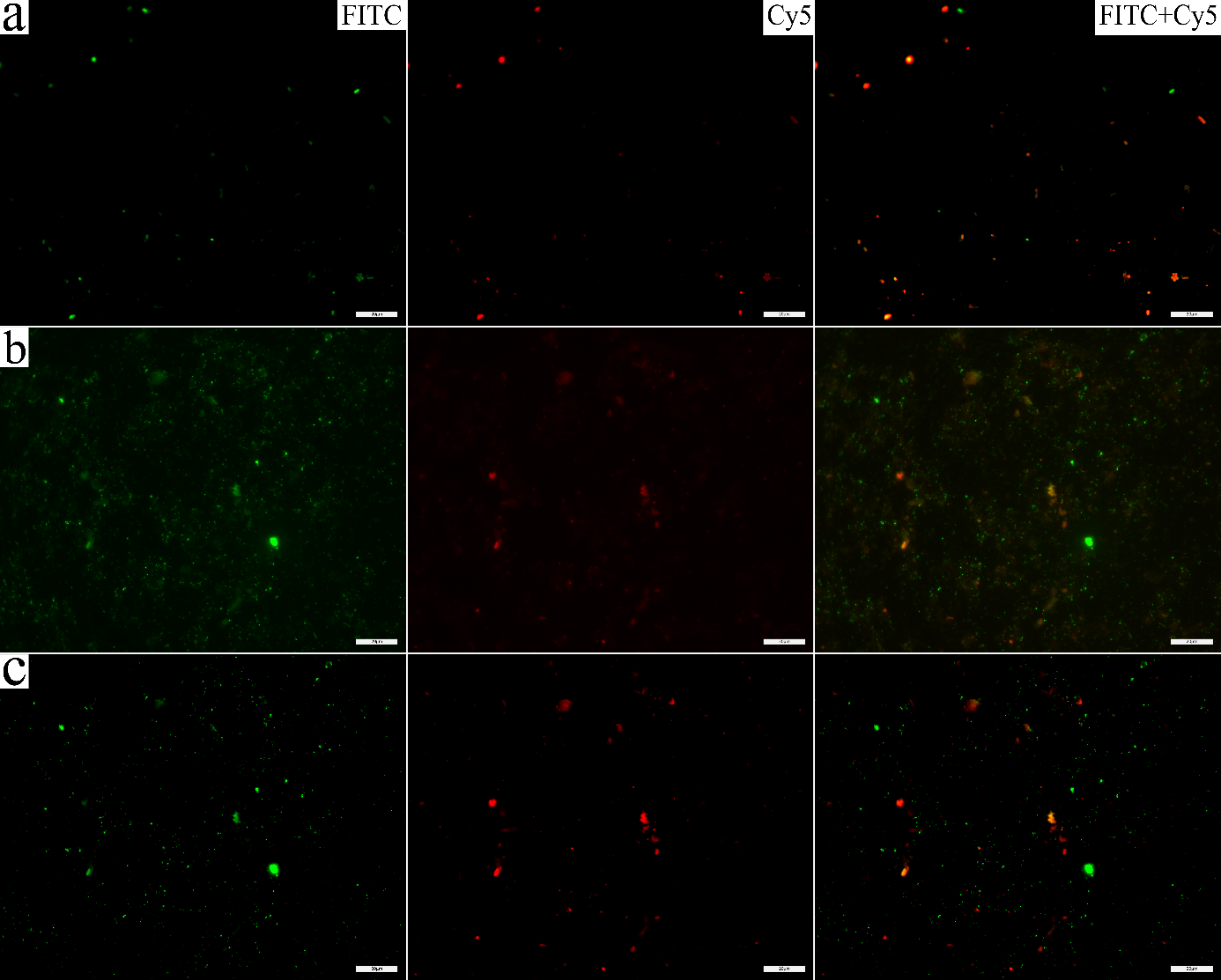 |
| --- |
| *Figure S6:* *nonEUB338****-****Cy5 as a negative control for phage signals during image enhancement.*  *a) F12 probed with EUB338-Cy3 (not shown), nonEUB338-Cy5 (red) and phage group E probes (no target, green). FITC spectrum shows only background noise and auto-fluorescence.*  *b) F49 probed with EUB338-Cy3 (not shown), nonEUB338-Cy5 (red), and phage group E probes (green). Cy5 signal overlaps with background signals but not with phage signals.*  *c) F49 probed with EUB338-Cy3 (not shown), nonEUB338-Cy5 (red), and phage group E probes (green). Cy5 signals are used as negative control as image is enhanced to reduce background noise.* |
