# Supplementary material for: Detection of specific uncultured bacteriophages by fluorescence in situ hybridisation in pig microbiome
Source: PLoS One. 2023 Mar 30;18(3):e0283676. doi: 10.1371/journal.pone.0283676 (PMC10062541; doi:10.1371/journal.pone.0283676)
Supplement: Attachment — Submitted filename: Response_to_Reviewers.docx [file pone.0283676.s003.docx]

Review Comments to the Author

Please use the space provided to explain your answers to the questions above. You may also include additional comments for the author, including concerns about dual publication, research ethics, or publication ethics. (Please upload your review as an attachment if it exceeds 20,000 characters)

Reviewer #1: General comments:

Ostenfeld et al presented certain novelty in the visualization of jumbophages from metagenome assembled genome, and made certain degree of improvements on the original FISH protocol for the special sample type. However, the whole structure is distractive by some unnecessary information, and the lay out also could be improved by moderate revision:

1. The phage plaque assay (from line 112 - 134) on E. coli cannot significantly contribute to the theme, also kinds of misleading as readers might be confused with the jumbophage’s host is E. coli. I would thus suggest remove this part to avoid the confusion.

“We thank the reviewer for their comment. We agree that this part could be confusing and should be improved. We have now adjusted the text and reduced it to briefly mention the *E. coli* assays only amongst other tested hosts (Lines: 114-138 – numbers are from the track change version). We believe that our plaque assays with *E. coli* as a host provide a valid evaluation step that jumbophages can be *in vitro* detected with plaque assay from those faecal samples. An in vitro detection of jumbophages adds value to the manuscript. However, we do not mention in the text that *E. coli* is the host to all jumbophages, we only mention that *E. coli* is potential host to those jumbophages that are isolated from the *E. coli* plaque assays as a validation step. This was also mentioned in lines 213-220 to avoid confusion.”

2. The sequenced phage contigs (Table S1) didn’t give much information here, only the contig length, is there any host-prediction work was done? According to the contig size, more details should be obtained by additional annotation. Besides, when phage contigs were assembled from 2 different samples, then based on which information that these two contigs were considered to be one? And when designing primers, which contig were used for each phage?

“We thank the reviewer for this comment. The purpose of providing the mentioned phage contigs in Table S1 is solely to show that those phages are of large size (as the reviewer mention in their comment). We refrain from carrying any further analyses on such sequencing output to leave those for an *in silico*-based studies (such as Al-Shayeb et al. that we used their published phage genomes to design our primers as mentioned in the text). The targeted genomes previously published were isolated from the Danish pig farms in questions. While predictions of bacteriophage hosts from *in silico* data can be complex, there are several online tools that are currently being developed to serve such purpose (PHERI, HostPhinder, Host Taxon Predicter, WIsH, MARVEL, Seeker, Phirbo, VirHostMatcher and others) using metagenomic sequencing from Illumina platform (which is the common practice). We therefore refrain from carrying out any of those predictions from our contig information in table S1, as the main purpose of the study is to develop an *in vitro* visualisation method to microscopically detect jumbophages (and *in vitro* detect them hence the *E. coli* host culturing in lines 213-220).
Regarding the phage contigs and how they were handled, we agree it was not clear from the manuscript and have now updated it accordingly. The phage complete and draft genome scaffolds were derived from the Al-Shayeb study, where analysis on whether these hundreds of large circular DNA entities were phage-like, plasmid-like and shared similarity to known sequences. The genomes used for primer design here were therefore much larger and often circular (200Kbp+ jumbo-phage genomes). Pairs of these previously published, high-identity genomes derived from different Danish pig farms were then aligned using progressive Mauve and inspected for especially conserved regions. Such high-identity genomic regions (>300bp) were visually identified, extracted and used as input for the version of Primer3 integrated in Geneious software. We looked for high-scoring primer pairs (20-30bp with a 25bp optimum), with 300bp product sizes and a Tm around 60C (57-60C). Several such pairs were identified for each target genome and the PCR probe products were then searched against NCBI nt using BlastN to ensure specificity. The text in the manuscript was adjusted accordingly (Lines 157-164).”

3. In Fig 3, the phage-host relation’s part is less convincing, as the phage clouds around bacteria, but no signal could be observed directly inside a bacterial cell, which is quite abnormal in other FISH publications. Some figure in supplementary material shows better results, may be worth to consider switch the position.

“We thank the reviewer for this suggestion. We chose this sample (F49) and its phageFISH images to be in the main text as it shows potential signals for various stages of phages: within the bacterial cells as an advanced stage of infection (red arrow in Fig S4 which is the original image for Fig 3 – and the top left arrow in the current Fig 3) and potentially early stages of infections (yellow arrow in Fig S4 which is the original image for Fig 3 – and the top right arrow in the current Fig 3). This also in agreement with the findings from Allers et al., 2013 showing various stages of phage infection with phageFISH, where phages were not only within the cells, but also surround the bacterial cells.”

Detailed suggestions:
Also, some small points that could be improved:

1. Line 153: what software used for primer design? Based on what parameters?

“The primers were designed using Primer3 v.2.3.7 in Geneious with mostly default parameters, other thans: primer length: 20-30, optimal primer length: 25, Tm: 57-63, product length: 300 bp. The text was adjusted accordingly in lines: 157-164. Please see the answer to the point above for more details on primer design and the sequences used.”

2. No signal could be observed for all the nonEUB338-Cy5 probe, in Fig 1 or Fig S4. As I could understand, this is a negative control probe, target on nothing. So, the description in line 189 about the overlapping signal with background is confusing, adding some arrows might be helpful.

“We thank the reviewer for this, we agree. We have now added an arrow to Fig 1 to indicate the targeted signal and adjusted the figure legend. Same to S5 and S6 Figs. We have also edited the text in that part of the main manuscript to make it clearer in lines: 202-209”

References:

Al-Shayeb B, Sachdeva R, Chen LX, et al. Clades of huge phages from across Earth’s ecosystems. *Nature*. 2020;578:425-431. doi:10.1038/s41586-020-2007-4
Allers E, Moraru C, Duhaime MB, et al. Single-cell and population level viral infection dynamics revealed by phageFISH, a method to visualize intracellular and free viruses. Environ Microbiol. 2013;15(8):2306-2318. doi:10.1111/1462-2920.12100
